# ILC2-derived LIF licences progress from tissue to systemic immunity

**DOI:** 10.1038/s41586-024-07746-w

**Published:** 2024-08-07

**Authors:** Mayuri Gogoi, Paula A. Clark, Ana C. F. Ferreira, Noe Rodriguez Rodriguez, Morgan Heycock, Michelle Ko, Jane E. Murphy, Victor Chen, Shi-Lu Luan, Helen E. Jolin, Andrew N. J. McKenzie

**Affiliations:** MRC Laboratory of Molecular Biology, Cambridge, UK

**Keywords:** Innate lymphoid cells, Lymph node, Leukaemia inhibitory factor, Mucosal immunology

## Abstract

Migration and homing of immune cells are critical for immune surveillance. Trafficking is mediated by combinations of adhesion and chemokine receptors that guide immune cells, in response to chemokine signals, to specific locations within tissues and the lymphatic system to support tissue-localized immune reactions and systemic immunity^1,2^. Here we show that disruption of leukaemia inhibitory factor (LIF) production from group 2 innate lymphoid cells (ILC2s) prevents immune cells leaving the lungs to migrate to the lymph nodes (LNs). In the absence of LIF, viral infection leads to plasmacytoid dendritic cells (pDCs) becoming retained in the lungs where they improve tissue-localized, antiviral immunity, whereas chronic pulmonary allergen challenge leads to marked immune cell accumulation and the formation of tertiary lymphoid structures in the lung. In both cases immune cells fail to migrate to the lymphatics, leading to highly compromised LN reactions. Mechanistically, ILC2-derived LIF induces the production of the chemokine CCL21 from lymphatic endothelial cells lining the pulmonary lymphatic vessels, thus licensing the homing of CCR7^+^ immune cells (including dendritic cells) to LNs. Consequently, ILC2-derived LIF dictates the egress of immune cells from the lungs to regulate tissue-localized versus systemic immunity and the balance between allergen and viral responsiveness in the lungs.

## Abstract (web summary)

Disruption of leukaemia inhibitory factor production from group 2 innate lymphoid cells prevents immune cells leaving the lungs to migrate to lymph nodes, leading to plasmacytoid dendritic cells becoming retained in the lungs following viral infection.

## Main

The lungs are constantly exposed to the inhalation of both infectious and non-infectious agents. The immune system must respond efficiently and appropriately to combat respiratory pathogens and/or repair tissue damage but also avoid inappropriate and potentially harmful inflammation such as allergic asthma^3^ or virus-induced pathology^4^. The emplacement of innate lymphoid cells (ILCs) as immune sentinels within mucosal surfaces allows them to survey tissues to help counter inappropriate immune reactions and maintain homeostasis, but also to react rapidly to protect against infection or injury^5–7^. This requires the orchestration of a highly dynamic system in which the circulation and homing of specialized immune cells must be coordinated in response to tissue-derived cues^3,5^.

During allergen-induced chronic type 2 lung inflammation, alarmin-like cytokines such as interleukin-33 (IL-33), IL-25 and thymic stromal lymphopoietin activate ILC2 proliferation and the vigorous production of type 2 effector cytokines (including IL-4, IL-5, IL-13 and amphiregulin) promoting T helper 2 (T_H_2) cell differentiation, immunoglobulin E (IgE) production, eosinophilia, mucus hypersecretion and airway contraction, but also aberrant tissue repair that can lead to fibrosis^3^. Lung ILC2s also have roles in the regulation of type 1 responses to infection by respiratory viruses including rhinovirus^8^, influenza virus^9,10^ and respiratory syncytial virus (RSV)^11^, with dysregulation associated experimentally with viral-induced asthma exacerbation^12^. Similarly, imbalanced pathogen-induced inflammation, as opposed to tissue repair, can lead to immune-mediated pathology. For example, infection with influenza A virus or severe acute respiratory syndrome coronavirus 2 (SARS-CoV-2) induces type I interferon production from pDCs, which may act antivirally or contribute to immunopathology^13,14^.

Cell migration and homing help control the differential magnitude of localized pulmonary tissue responses versus the dissemination of systemic immune responses following allergen or virus exposure^15–19^. Indeed, following the disruption of the CCL21 chemokine–CCR7 chemokine receptor pathway, rather than DCs and antigen-specific T cells and B cells migrating to the secondary lymphoid organs (lymph nodes, LNs) to expand before returning to the inflamed tissues, they remain in the lung to develop tertiary lymphoid structures (TLS) in the form of inducible bronchus-associated lymphoid tissues (iBALT). This tissue-localized inflammation can lead to enhanced antiviral immunity^19^ but is also associated with chronic allergic responses^15–18,20^. Here we investigated how ILC2s can modify the lung microenvironment to promote systemic immunity and counterbalance tissue-localized antiviral responses.

## ILC2s are required for normal homing of pDCs

**Fig. 1:**
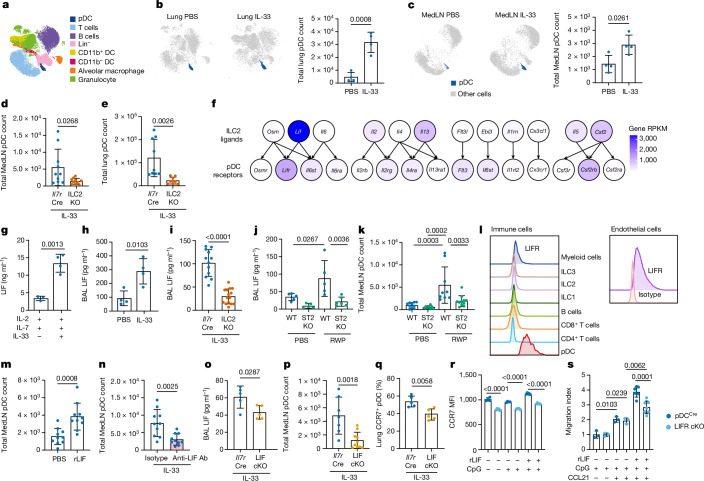
ILC2s are required for normal homing of pDCs. **a**–**c**, Uniform manifold approximation and projection of lung and MedLN immune cell clusters (**a**) and quantification of pDCs (**b**,**c**) from flow cytometry analysis following PBS and IL-33 intranasal treatment of lung (**b**) and MedLN (**c**) in WT mice (*n* = 4). **d**,**e**, Flow cytometry analysis of *Il7r*^Cre^ and *Il7r*^Cre^*Rora*^flox/flox^ pDCs in MedLN (**d**, *n* = 10) and lung (**e**, *n* = 9). **f**, Heatmap of ligand and receptor expression derived from RNA-seq data. **g**–**j**, ELISA of LIF from purified ILC2s cultured with IL-2 + IL-7, with (*n* = 4) or without (*n* = 3) IL-33 (**g**); BAL of mice treated with IL-33 (**h**, WT, *n* = 4; **i**, *Il7r*^Cre^, *n* = 12; ILC2KO, *n* = 15); and with either ragweed protein (RWP, **j**, WT, *n* = 5; ST2KO, *n* = 6) or PBS (WT, *n* = 5; ST2KO, *n* = 5). **k**, Flow cytometry analysis of pDCs (WT: PBS, *n* = 10; RWP, *n* = 9; ST2KO: PBS, *n* = 8; RWP, *n* = 11). **l**, Flow cytometry analysis of LIF receptor (LIFR) on indicated cell types. **m**,**n**, Flow cytometry analysis of pDCs in WT mice following rLIF treatment (**m**, *n* = 10) and anti-LIF neutralizing antibody (Ab) and IL-33 treatment (**n**, *n* = 10). **o**, ELISA of BAL LIF following IL-33 challenge (*n* = 5). **p**,**q**, Flow cytometry analysis of pDCs in *Il7r*^Cre^ (*n* = 7) and LIF-cKO (*n* = 10) (**p**), and lung CCR7^+^ pDC (**q**) percentage (*n* = 5), following IL-33 challenge. **r**, Flow cytometry analysis of CCR7 expression by CpG-activated pDC^Cre^ and LIFR-cKO pDCs with or without rLIF for 16 h (pDC^Cre^, *n* = 4; pDC^Cre^ + CpG, *n* = 4; pDC^Cre^ + CpG + rLIF, *n* = 5; LIFR-cKO, *n* = 5; LIFR-cKO + CpG, *n* = 5; LIFR-cKO + CpG + rLIF, *n* = 5). **s**, Chemotaxis of CpG-activated pDC^Cre^ or LIFR-cKO pDCs to CCL21 with or without rLIF for 16 h (pDC^Cre^ + CpG, *n* = 3; pDC^Cre^ + CpG + CCL21, *n* = 3; pDC^Cre^ + CpG + rmLIF + CCL21, *n* = 7; LIFR-cKO + CpG, *n* = 3; LIFR-cKO + CpG + CCL21, *n* = 3; LIFR-cKO + CpG + rmLIF + CCL21, *n* = 7). **b**–**e,g**–**i,m**–**s**, Unpaired two-sided *t*-test; **j**,**k**, one-way analysis of variance (ANOVA) with Tukey’s multiple-comparisons test; data (mean ± s.e.m.) are either representative (**a**–**c**,**g**,**h**,**j**,**l**,**o**,**q**–**s**) or pooled from two independent experiments (**d**,**e**,**i**,**k**,**m**,**n**,**p**). Lineage (Lin) contains CD3, CD4, CD8a, CD19, CD11b, CD11c and FcER1 antibodies; RPKM, reads per kilobase per million mapped reads. Source Data

**Extended Data Fig. 1:**
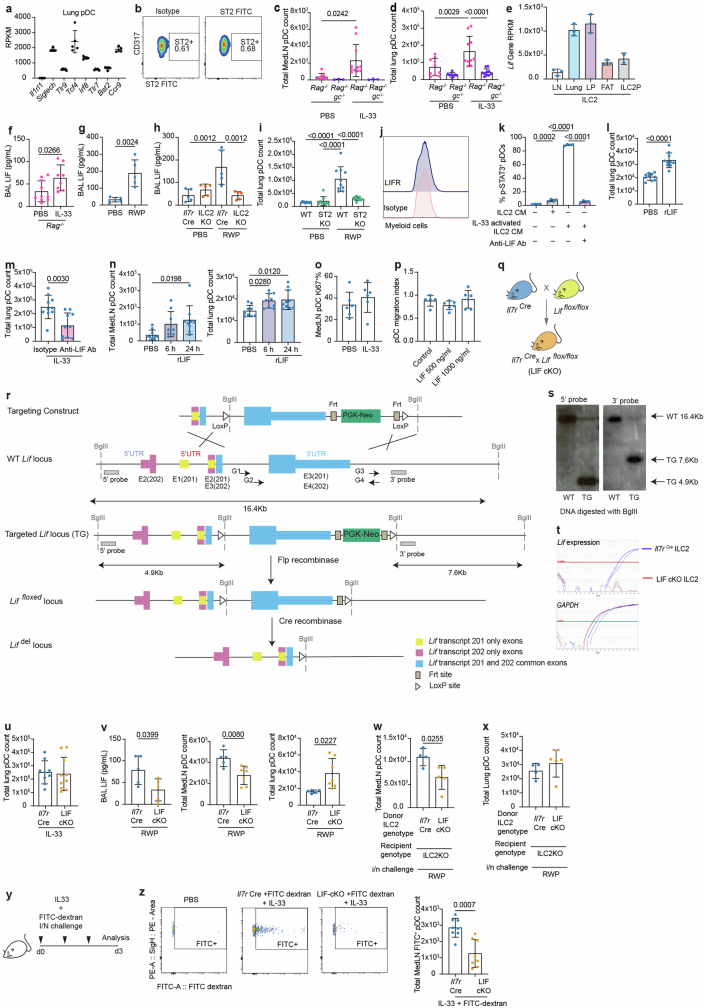
ILC2 production of LIF during type-2 immune response. **a**, Gene expression of indicated genes in pDCs from bulk RNAseq data with five biological replicates per group. **b**. Representative flow cytometry plots of lung pDCs stained for ST2. Flow cytometry analysis of pDC numbers in **c**, MedLN, *Rag2*^–/–^ (PBS = 8, IL-33 = 11) and *Rag2*^–/–^*Il2rgc*^–/–^ (*Rag2*^–/–^*gc*^–/–^) (PBS = 3, IL-33 = 3), and **d**, lung *Rag2*^–/–^ (PBS = 10, IL-33 = 10) and *Rag2*^–/–^*gc*^–/–^ (PBS = 8, IL-33 = 10) following IL-33 intranasal challenge. **e**, *Lif* expression in ILC2 purified from lymph node (LN) (n = 3), lung (Lung) (n = 3), small intestine lamina propria (LP) (n = 3), adipose tissue (FAT) (n = 3), bone marrow ILC2 progenitor (ILC2P) (n = 2) from naïve mice. ELISA of LIF in the BAL **f**, from *Rag2*^–/–^ (PBS = 10, IL-33 = 8) mice following IL-33 intranasal challenge, and **g**, from wildtype mice (WT) following ragweed protein extract (RWP) challenge (PBS = 5, RWP = 5), and from **h**, *Il7r*^Cre^ (PBS = 5, RWP = 5) and *Il7r*^Cre^ x *Rora*^flox/flox^ (ILC2KO) (PBS = 5, RWP = 5) mice treated with RWP. **i**, Flow cytometry analysis of lung pDCs in WT (PBS = 10, RWP = 10 IL-33 receptor-deficient (ST2KO) (PBS = 9, RWP = 11) mice following RWP intranasal challenge. **j**, Flow cytometry analysis of LIF receptor (LIFR) staining on lung myeloid cells. Flow cytometry analysis of **k**, phospho-STAT3 positive cells following activation as indicated, ILC2 conditioned media (ILC2 CM), IL-33 activated ILC2 conditioned media (IL-33 activated ILC2 CM), anti-LIF neutralizing antibody (n = 4), and **l**, lung pDCs following rLIF treatment in wildtype mice (n = 10), and **m**, lung pDCs in wild type mice following treatment with anti-LIF neutralizing antibody or isotype and IL-33 intranasal challenge (n = 10), and **n**, pDCs in the MedLN (PBS = 10, 6 h = 9, 24 h = 9) and lung (PBS = 9, 6 h = 9, 24 h = 10) at the indicated timepoints following one dose of rLIF challenge, and **o**, MedLN pDC Ki67 expression in wild type mice following IL-33 challenge (n = 6). **p**, Trans-well chemotaxis of pDCs to rLIF. (control = 5, LIF (500 ng/ml) = 6, LIF (1000 ng/ml) = 6). **q**, *Il7r*^Cre^ x *Lif*^flox/flox^ (LIF-cKO) generation schematic. **r**, Schematic of *Lif*^flox/flox^ mouse generation. **s**, Southern analysis of *Lif*^flox/flox^ ES cells. **t**, qPCR analysis of *Lif* expression in ILC2s purified from *Il7r*^*Cre*^ and *Il7r*^*Cre*^ x *Lif*^flox/flox^ (LIF-cKO) mice. **u**, Flow cytometry analysis of lung pDC numbers in *Il7r*^*Cre*^ (n = 9) and LIF-cKO (n = 10) following IL-33 challenge. **v**, ELISA of BAL LIF from RWP challenged *Il7r*^Cre^ and LIF-cKO mice (*Il7r*^Cre^ = 5 and LIF-cKO =5), and flow cytometry analysis of MedLN pDCs (*Il7r*^Cre^ = 5 and LIF-cKO =7) and lung pDCs (*Il7r*^Cre^ = 5 and LIF-cKO =7). Flow cytometry analysis of **w**, MedLN, and **x**, lung pDCs in recipient ILC2 KO mice transplanted with *Il7r*^*Cre*^ or LIF-cKO ILC2s and subsequently challenged with RWP. (*Il7r*^Cre^ = 4 and LIF-cKO =5). **y**, Schematic of mouse FITC-dextran and IL-33 intranasal challenge. **z**, Flow cytometry gating strategy and MedLN FITC-dextran^+^ pDCs numbers in *Il7r*^Cre^ and LIF-cKO mice following PVM and FITC-dextran intranasal challenge (*Il7r*^Cre^ = 8 and LIF-cKO =8). **f-h,m-p,u-x,z**, unpaired two-sided *t*-test, **c,d,i,k,l** one-way ANOVA with Tukey’s multiple comparisons test. Data mean ± SEM. Data are representative of two (b, g, h, j, k, o, p, v, w, x) independent experiments with similar results. Experiments in (c, d, f, i, l, m, n, u, z) are pooled data from two independent experiments. RNA-seq data in a are based on five biological replicates per group. Source Data

Using multiparametric flow cytometry to characterize rapid immune cell changes in the lung during the onset of a type 2 immune response induced by acute treatment with recombinant mouse IL-33 (refs. ^21,22^), we noted an unexpected increase in CD45^+^CD317^+^Siglec-H^+^F4/80^−^CD11b^−^ pDCs in the lung and the lung-draining mediastinal LN (MedLN) (Fig. 1a–c). pDCs develop in the bone marrow and are key producers of antiviral type I IFN, which drives innate antiviral responses^23^. During inflammation, pDCs upregulate chemokine receptors and are rapidly recruited from the circulation into the spleen and peripheral LNs (CCR7-, CXCR3- and CCR5-mediated)^23^ or intestinal tissue (CCR9-mediated)^24^. Although gene expression analysis confirmed the phenotype of lung pDCs (Extended Data Fig. 1a), these did not express the IL-33 receptor (ST2, *Il1rl1* gene; Extended Data Fig. 1a,b), suggesting an indirect mechanism by which IL-33 induces pDCs. By treating T cell- and B cell-deficient (*Rag2*^−/−^) mice or lymphocyte-deficient (*Rag2*^−/−^*Il2rg*^−/−^) mice with IL-33, we determined that ILCs are required for the IL-33-induced pDC increase but that T cells and B cells are not (Extended Data Fig. 1c,d). IL-33-treated, ILC2-deficient (ILC2KO, *Il7r*^Cre^*Rora*^flox/flox^) mice also failed to induce pDCs, indicating a role for ILC2s in pDC regulation (Fig. 1d,e).

## ILC2-derived LIF regulates pDC migration to LNs

For identification of potential ILC2-derived regulators of pDC biology we cross-referenced cytokine receptors expressed by pDCs with cytokine ligands produced by ILC2s. LIF, produced by ILC2s, and LIF receptor (LIFR), expressed by pDCs, represented attractive targets (Fig. 1f) because the role of ILC2-derived LIF is unknown and LIF can negatively regulate pDC development and type I IFN production^25,26^. LIF is a pleiotropic cytokine with roles in embryonic development and promotion of tumorigenesis, but its functions in immunity are understudied^27^. *Lif* was expressed by ILC2s from several tissues (Extended Data Fig. 1e), and LIF protein was produced by in vitro cultured ILC2s (Fig. 1g) and in vivo in bronchoalveolar lavage (BAL) in response to IL-33 (Fig. 1h), independently of T lymphocytes and B lymphocytes (Extended Data Fig. 1f) but requiring ILC2s (Fig. 1i). Short-term administration of ragweed pollen (RWP) extract, an inducer of ILC2-dependent allergic immune reactions in the lung^21^, also upregulated LIF production (Extended Data Fig. 1g), which required ILC2s (Extended Data Fig. 1h) and signalling via the ST2 receptor (assessed using ST2-deficient mice) correlating with fewer pDCs in the MedLN (Fig. 1j,k and Extended Data Fig. 1i).

Next we determined the expression of LIFR on lung cells. Little LIFR was observed on ILC1s, ILC2s, ILC3s, T cells, B cells or myeloid cells (Fig. 1l and Extended Data Fig. 1j) but was present on pDCs and CD31^+^ endothelial cells (Fig. 1l). Bone marrow-derived pDCs also responded to ILC2-derived LIF as determined by their phosphorylation of STAT3 (p-STAT3), a key signalling component of the LIFR signalling pathway^25,26^, which was ablated by the inclusion of anti-LIF neutralizing antibody (Extended Data Fig. 1k). Furthermore, intranasal administration of recombinant LIF (rLIF) induced pDCs in both MedLN and lung (Fig. 1m and Extended Data Fig. 1l) whereas anti-LIF neutralizing antibody inhibited pDC induction following IL-33 treatment in vivo (Fig. 1n and Extended Data Fig. 1m). The increase in pDCs occurred within 6 h (Extended Data Fig. 1n) in the absence of changes in pDC proliferation (Extended Data Fig. 1o), suggesting that pDC accumulation had resulted from recruitment rather than cell division. However, LIF did not induce pDC migration directly (Extended Data Fig. 1p).

For investigatation of the roles of ILC2-derived LIF we generated *Il7r*^Cre^*Lif*^flox/flox^ (LIF-cKO) mice to delete LIF in lymphocytes (Extended Data Fig. 1q–t). Intranasal administration of IL-33 to LIF-cKO mice resulted in reduced LIF concentrations in BAL (Fig. 1o) and fewer pDCs in the MedLN (Fig. 1p). However, lung pDCs were not reduced (Extended Data Fig. 1u), in contrast to the reduction observed in ILC2-deficient mice, suggesting that additional ILC2-dependent factors may also be in play. Similar results were obtained from RWP challenge of LIF-cKO mice (Extended Data Fig. 1v). Interestingly, although lymphocyte-derived LIF did not account for all LIF detected in the BAL, other sources of LIF were not capable of rescuing the observed phenotype. This suggests a more localized action for lymphocyte-derived LIF, such as in stromal cell niches within lung perivascular adventitial cuffs in which tissue-resident ILC2s are known to be present^28^. For assessment of the role of ILC2-derived LIF, we transferred either purified LIF-producing ILC2s or LIF-deficient ILC2s into ILC2KO mice and then challenged them with RWP. Transfer of LIF-producing ILC2s resulted in more pDCs in MedLNs than induced by LIF-deficient ILC2s (Extended Data Fig. 1w). There was no difference in lung pDC numbers (Extended Data Fig. 1x). Thus, ILC2-derived LIF is sufficient to enhance pDC numbers in MedLNs. For visualization of the defect in the migration of pDCs from the lung to MedLN we delivered fluorescein isothiocyanate (FITC)-dextran intranasally into the lungs of mice and assessed the migration of FITC-labelled pDCs to the MedLN in response to IL-33 lung challenge (Extended Data Fig. 1y). LIF-cKO mice showed reduced migration of FITC-dextran-positive pDCs from the lung to the MedLN as compared with controls (Extended Data Fig. 1z), demonstrating LIF-mediated regulation of pDC migration.

**Extended Data Fig. 2:**
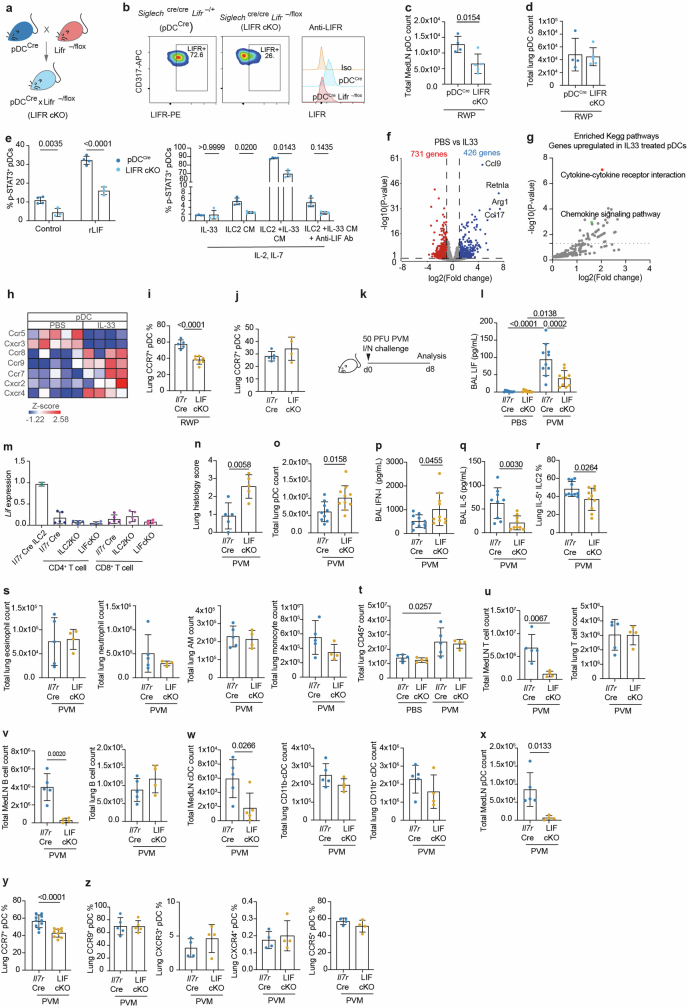
pDC expression of LIFR and Pneumovirus infection of conditional LIF-deficient mice. **a**, Schematic of pDC-specific LIF receptor (LIFR) deficient pDC^Cre^ x *Lifr* ^–/flox^ (LIFR cKO) mice generation. **b**, LIF receptor (LIFR) expression in lung pDCs in *Siglech*^Cre/Cre^ x *Lifr* ^–/+^ (pDC^Cre^) and *Siglech*^Cre/Cre^ x *Lifr* ^–/flox^ (LIFR cKO) mice. Flow cytometry analysis of pDC^Cre^ and LIFR cKO **c**, MedLN, and **d**, lung pDCs following RWP challenge (pDC^Cre^ =4 and LIFR cKO = 5). **e**, Flow cytometry analysis of phospho-STAT3 positive cells following activation as indicated, ILC2 conditioned media (ILC2 CM), IL-33 activated ILC2 conditioned media (IL-33 + ILC2 CM), anti-LIF neutralizing antibody (control pDC^Cre^ = 4, control LIFR cKO =3, rLIF pDC^Cre^ = 4, rLIF LIFR cKO =3, IL-33 = 4, ILC2 CM = 4, IL-33 + ILC2 CM = 4, IL-33 + ILC2 CM+Anti-LIF Ab =4). **f**, Volcano plot of all differentially expressed genes in bulk RNAseq of lung pDCs from PBS or IL-33 challenged wildtype mice. **g**, KEGG pathway analysis of significant differentially expressed genes from (**f**). **h**, Heatmap of significant differentially expressed chemokine receptor genes from lung pDC bulk RNAseq. Flow cytometry analysis of lung **i**, CCR7^+^pDCs in LIF-cKO mice after RWP challenge (*Il7r*^Cre^ = 5 and LIF-cKO =7), and **j**, CCR7^+^pDCs in naïve LIF-cKO mice (*Il7r*^Cre^ = 5 and LIF-cKO =4). **k**, schematic of the experimental protocol for pneumovirus of mouse (PVM) infection. **l**, ELISA of LIF in the BAL from *Il7r*^Cre^ and LIF-cKO mice following PVM challenge (n = 10). **m**, qPCR analysis of *Lif* gene expression in ILC2s, CD4^+^ and CD8^+^ T cells purified from indicated mice following PVM intranasal challenge. *Il7r*^*Cre*^, ILC2KO and LIF-cKO mice (*Il7r*^*rer*^ ILC2 = 2, *Il7r*^*Cre*^ CD4^+^T = 5, *Il7r*^*Cre*^ CD8^+^ T = 4, ILC2KO CD4^+^T =4, ILC2KO CD8^+^ T = 5, LIF-cKO CD4^+^T =4, LIF-cKO CD8^+^ T = 5). **n**, Lung histology infiltration score of *Il7r*^Cre^ and LIF-cKO mice following PVM challenge (*Il7r*^Cre^ = 5 and LIF-cKO =5). **o**, Flow cytometry analysis of lung pDCs in *Il7r*^Cre^ and LIF-cKO mice following PVM challenge (*Il7r*^Cre^ = 9 and LIF-cKO =10). ELISA of: **p**, IFN-I in BAL (*Il7r*^Cre^ = 10 and LIF-cKO =10);and **q**, IL-5 in BAL (*Il7r*^Cre^ = 9 and LIF-cKO =9) from *Il7r*^Cre^ and LIF-cKO mice following PVM challenge. **r**, Flow cytometry analysis of lung IL-5^+^ILC2s in lung in *Il7r*^Cre^ and LIF-cKO mice after PVM challenge (*Il7r*^Cre^ = 10 and LIF-cKO =10). **s**, Flow cytometry analysis of MedLN and lung eosinophil, neutrophil, alveolar macrophage (AM) and monocyte numbers following PVM challenge (*Il7r*^Cre^ = 5 and LIF-cKO =4). Flow cytometry analysis of **t**, lung CD45^+.^cell numbers *Il7r*^Cre^ (PBS = 5, PVM = 5) and LIF-cKO (PBS = 5, PVM = 4); **u**, T cell numbers; **v**, B cells; **w**, cDC; **x**, pDCs from MedLN and lung from *Il7r*^Cre^ and LIF-cKO mice following PVM challenge (*Il7r*^Cre^ = 5 and LIF-cKO =4). **y**, Flow cytometry analysis of lung pDCs for CCR7 expression in *Il7r*^*Cre*^ and LIF-cKO mice following PVM challenge (*Il7r*^Cre^ = 10 and LIF-cKO =11). **z**, Flow cytometry analysis of lung pDCs for CCR9 (*Il7r*^Cre^ = 5 and LIF-cKO =5), CXCR3 (*Il7r*^Cre^ = 4 and LIF-cKO =4), CXCR4 (*Il7r*^Cre^ = 4 and LIF-cKO =4), and CCR5 (*Il7r*^Cre^ = 4 and LIF-cKO =4) in *Il7r*^*Cre*^ and LIF-cKO mice following PVM challenge. **c,d,i,j,n-s,u-z** unpaired two-sided *t*-test, **e,l,t** one-way ANOVA with Tukey’s multiple comparisons test. Data mean ± SEM. Data are representative of two (b, c, d, e, l, i,j,m,n,s-x,z) independent experiments with similar results. Experiments in (l,o-r,y) are pooled data from two independent experiments. RNA-seq data in a are based on five biological replicates per group. Source Data

We also investigated the reciprocal part played by LIFR expression on pDCs by intercrossing pDC^Cre^ (Tg^Siglech-Cre,-mCherry^) mice with *Lifr*^−/flox^ mice (Extended Data Fig. 2a). Despite inefficient LIFR deletion from pDCs (Methods and Extended Data Fig. 2b), pDC^Cre^*Lifr*^−/flox^ mice showed impaired pDC induction in MedLNs (but not in lungs) following RWP challenge as compared with controls (Extended Data Fig. 2c,d), mirroring mice lacking LIF production from lymphocytes. Bone marrow-derived pDCs from pDC^Cre^*Lifr*^−/flox^ mice also showed reduced STAT3 phosphorylation as compared with control (Extended Data Fig. 2e). Thus ILC2-derived LIF is produced in response to IL-33 and allergen challenge and regulates the proportions of pDCs in both lung tissue and MedLN.

## LIF promotes CCR7 expression on pDCs

For identification of cell migration-related factors induced in pDC by IL-33, we re-examined the RNA sequencing (RNA-seq) data from pDCs purified from the lungs of wild-type (WT) mice challenged intranasally with PBS or IL-33 (Extended Data Fig. 2f). The ‘cytokine–cytokine receptor interaction and chemokine signalling’ pathway was identified as the top hit from Kyoto Encyclopedia of Genes and Genomes (KEGG) pathway analysis of all genes with significant differential expression (Extended Data Fig. 2g). Notably, the chemokine receptor CCR7 was upregulated by IL-33 treatment (Extended Data Fig. 2h). CCR7 is important for lymphocyte migration into LNs^29^ and is also upregulated on pDCs following activation; CCR7-deficient pDCs show markedly impaired homing to LNs^30^. We found fewer CCR7^+^ pDCs in the lungs of LIF-cKO mice challenged intranasally with either IL-33 or RWP (Fig. 1q and Extended Data Fig. 2i,j). Notably, bone marrow-derived pDCs (which in our cultures were CCR7^+^ before CpG activation) showed that LIFR-deficient pDCs expressed less CCR7 (Fig. 1r) and evidenced reduced migration towards CCL21 (a CCR7 ligand) in the presence of rLIF compared with controls (Fig. 1s), although this may have been underestimated due to inefficient LIFR deletion from pDCs (Methods and Extended Data Fig. 2b). These data support a role for LIF-induced CCR7 expression on pDCs in their recruitment to MedLN.

## LIF promotes virus-induced CCR7^+^ cell migration

**Fig. 2:**
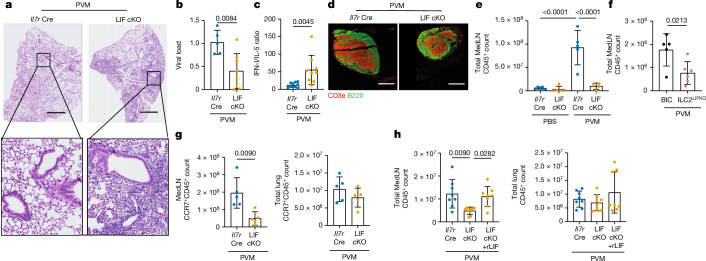
ILC2-derived LIF is required for all CCR7^+^ immune cell egress to LNs. **a**, Lung histology from *Il7r*^Cre^ and LIF-cKO mice following PVM challenge at day 8. **b**, Lung viral load following PVM challenge in *Il7r*^Cre^ (*n* = 5) and LIF-cKO (*n* = 8) mice. **c**, BAL type I IFN (IFN-I):IL-5 ratio from ELISA data following PVM challenge by *Il7r*^Cre^ (*n* = 10) and LIF-cKO (*n* = 9) mice. **d**, Histology of *Il7r*^*Cre*^ and LIF-cKO mouse MedLN following PVM challenge. B cells were stained with B220 antibody (green), T cells with CD3e antibody (red). **e**,**f**, Flow cytometry analysis of CD45^+^ cell numbers following PVM challenge in *Il7r*^Cre^ (PBS, *n* = 5; PVM, *n* = 5) and LIF-cKO (PBS, *n* = 5; PVM, *n* = 4) mice (**e**) and in BIC (*n* = 5) and BIC × *Lif*^flox/flox^ (ILC2^LIFKO^, *n* = 6) mice (**f**). **g**, Flow cytometry analysis of CCR7^+^CD45^+^ cell numbers following PVM challenge in *Il7r*^Cre^ and LIF-cKO mice (*n* = 5). **h**, CD45^+^ cell numbers in *Il7r*^*Cre*^ and LIF-cKO mice treated with rLIF and infected with PVM (*Il7r*^Cre^ + PVM, *n* = 8; LIF-cKO + PVM, *n* = 8; LIF-cKO + PVM + rLIF, *n* = 8). **b**,**c**,**f**,**g** Unpaired two-sided *t*-test; **e**,**h**, one-way ANOVA with Tukey’s multiple-comparisons test. Data presented as mean ± s.e.m. **a**,**d**–**g**, Data are representative of two independent experiments with similar results. **b**,**c**,**h**, Experiments are pooled data from two independent experiments. Scale bars, 1,000 µm (**a**), 200 µm (**d**). Source Data

**Extended Data Fig. 3:**
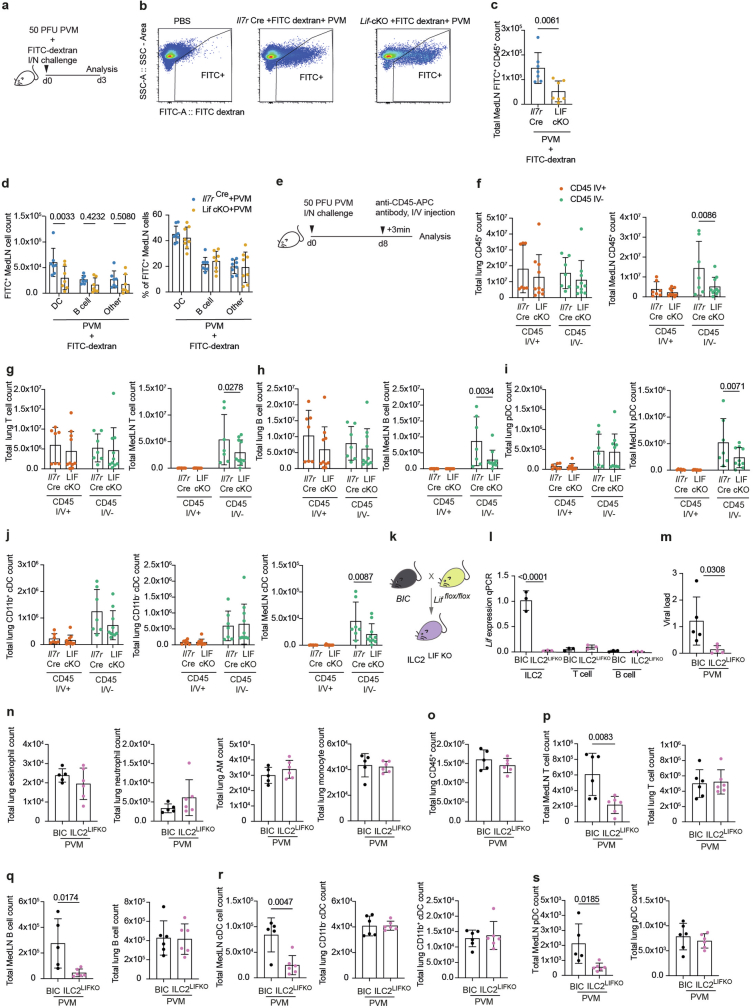
Assessment of circulating and tissue-resident immune cells in conditional LIF-deficient mice during PVM infection. **a**, Schematic of FITC-dextran and PVM intranasal challenge. **b**, Flow cytometry gating strategy and FITC-dextran^+^ CD45^+^ MedLN cell numbers in *Il7r*^Cre^ and LIF-cKO mice following PVM and FITC-dextran intranasal challenge. Flow cytometry analysis of: **c**, MedLN FITC-dextran^+^ cell numbers in *Il7r*^Cre^ and LIF-cKO mice following PVM and FITC-dextran intranasal challenge (*Il7r*^Cre^ = 7 and LIF-cKO =7); **d**, MedLN FITC-dextran^+^ DCs, B cells, and other immune cell numbers (*Il7r*^Cre^ = 7 and LIF-cKO =7) and percentages in *Il7r*^Cre^ and LIF-cKO mice following PVM and FITC-dextran intranasal challenge (*Il7r*^Cre^ = 8 and LIF-cKO =8). **e**, Schematic of intravenous anti-CD45-APC antibody labelling after PVM infection. Flow cytometry analysis of lung and MedLN intravenous anti-CD45-APC antibody labelled: **f**, CD45^+^ immune cells, **g**, T cells, **h**, B cells, **i**, pDCs, and **j**, cDCs after PVM infection (*Il7r*^Cre^ = 7 and LIF-cKO =10). **k**, ILC2^LIFKO^ generation schematic. **l**, qPCR analysis of *Lif* expression in lung ILC2, T cells and B cells purified from Boolean Cre (BIC) and BIC x *Lif*^flox/flox^ (ILC2^LIFKO^) mice after PVM challenge (BIC = 3, ILC2^LIFKO^ = 3). **m**, Lung viral load BIC and ILC2^LIFKO^ mice following PVM challenge (BIC = 5, ILC2^LIFKO^ = 5). **n**, Flow cytometry analysis of lung eosinophil (BIC = 5, ILC2^LIFKO^ = 5), neutrophils (BIC = 5, ILC2^LIFKO^ = 6), alveolar macrophages (AM) (BIC = 5, ILC2^LIFKO^ = 6) and monocytes (BIC = 5, ILC2^LIFKO^ = 6) in BIC and ILC2^LIFKO^ mice following PVM intranasal challenge. Flow cytometry analysis of: **o**, lung CD45^+^ cells (BIC = 5, ILC2^LIFKO^ = 6); **p**, T cells from MedLN (BIC = 6, ILC2^LIFKO^ = 6) and lung (BIC = 6, ILC2^LIFKO^ = 6); **q**, B cells from MedLN (BIC = 5, ILC2^LIFKO^ = 6) and lung (BIC = 6, ILC2^LIFKO^ = 6); **r**, cDCs from MedLN (BIC = 5, ILC2^LIFKO^ = 6) and lung (BIC = 6, ILC2^LIFKO^ = 6); **s**, pDCs from MedLN (BIC = 5, ILC2^LIFKO^ = 6) and lung (BIC = 6, ILC2^LIFKO^ = 6). **c,d,f-j,l-s** unpaired two-sided *t*-test. Data mean ± SEM. Data are representative of two (**b, l,m-s**) independent experiments with similar results. **c,d,f-j** unpaired two-sided *t*-test. Data mean ± SEM. Data are pooled from two independent experiments. Source Data

Given the importance of pDCs in immune responses to viruses, we next used pneumovirus of mice (PVM), a natural pathogen of mice that replicates in the respiratory tract (Extended Data Fig. 2k) and that can be used to model many of the pathological features of human RSV infection, including potent type I IFN production^31,32^. Eight days after PVM infection, increased LIF was detected in the BAL of control mice compared with LIF-cKO mice (Extended Data Fig. 2l), with lung ILC2s representing the predominant source of *Lif* as compared with T cells (Extended Data Fig. 2m). Notably, the lungs of PVM-infected LIF-cKO mice showed more virus-induced inflammatory cell aggregates (Fig. 2a and Extended Data Fig. 2n), which was accompanied by improved antiviral immunity as indicated by a reduced viral load (Fig. 2b). There was little increase in lung pDCs in LIF-cKO mice (Extended Data Fig. 2o and see below); however, there was an elevation in type I IFN in BAL (Extended Data Fig. 2p) suggesting that, by deletion of LIF, pDCs were no longer being inhibited thus resulting in greater type I IFN production and a reduction in IL-5 (Fig. 2c and Extended Data Fig. 2p–r), aligning with the potential of type I IFN to inhibit ILC2s^9,10^. However, lung eosinophils, neutrophils, alveolar macrophages and monocytes were unchanged (Extended Data Fig. 2s). By stark contrast, lung-draining MedLNs were notably smaller in the absence of LIF following viral challenge (Fig. 2d), which was associated with a profound deficit in all CD45^+^ immune cells in the MedLN but not in the lung (Fig. 2e and Extended Data Fig. 2t). This included T cells, B cells, conventional dendritic cells (cDCs) and pDCs as compared with controls (Extended Data Fig. 2u–x). Similar to the results observed in IL-33 and RWP challenge, PVM infection also resulted in a smaller proportion of pDCs expressing CCR7 (but not other chemokine receptors) in the absence of LIF (Extended Data Fig. 2y,z). For visualization and corroboration of the defect in cell migration, we labelled lung immune cells with FITC-dextran and assessed their homing to MedLN following lung challenge with PVM (Extended Data Fig. 3a). LIF-cKO mice showed reduced migration of FITC-labelled immune cells (predominantly DCs) to the MedLN compared with controls (Extended Data Fig. 3b–d). Furthermore, by intravenous administration of anti-CD45-APC antibody to label circulating blood cells during PVM infection, we confirmed that the LIF-dependent immune cell deficit in the MedLN had resulted from their impaired migration from the lungs via the lymphatic system, although the magnitude of the immune response to infection was smaller in these experiments, leading to more modest differences between LIF-cKO mice and controls (Extended Data Fig. 3e–j).

**Extended Data Fig. 4:**
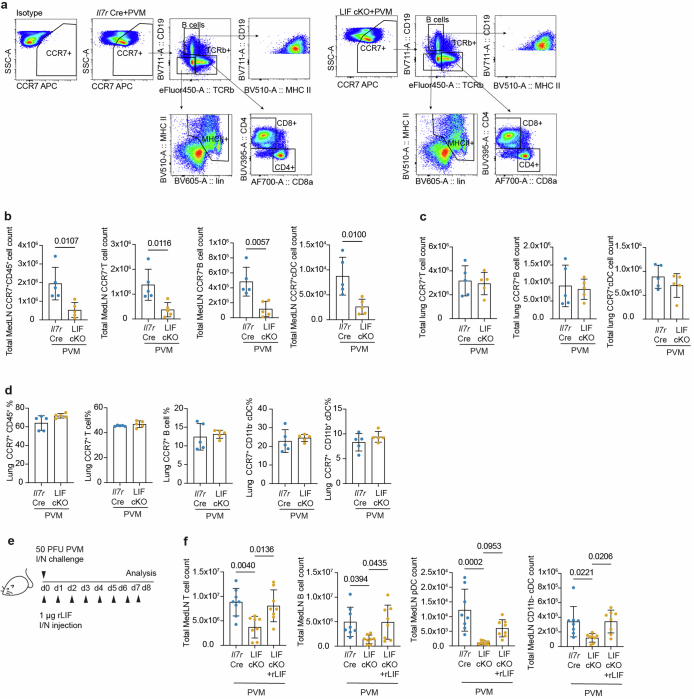
CCR7-positive immune cell analysis in conditional LIF-deficient mice. **a**, Flow cytometry gating strategy for CCR7^+^ CD45^+^ cells in *Il7r*^*Cre*^ and LIF-cKO mice following PVM challenge. **b-d**, *Il7r*^*Cre*^ and LIF-cKO mice following PVM challenge (*Il7r*^Cre^ = 5 and LIF-cKO =5). **b**, Flow cytometry analysis of CCR7^+^ cell numbers in MedLN; **c**, Flow cytometry analysis of CCR7^+^ cell numbers in lung. **d**, Flow cytometry analysis of CCR7^+^ cell percentages. **e**, Schematic of PVM infection combined with PBS or rLIF intranasal treatment. **f**, Flow cytometry analysis of MedLN T cells, B cells, pDCs and cDCs in *Il7r*^*Cre*^ and LIF-cKO mice treated with rLIF and infected with PVM (*Il7r*^Cre^ = 8 and LIF-cKO =8). **b-d**, unpaired two-sided *t*-test, **f**, one-way ANOVA with Tukey’s multiple comparisons test. Data mean ± SEM. Data are representative of two (a, b, c, d) independent experiments with similar results. Experiment (f) are pooled data from two independent experiments. Source Data

To confirm the specific requirement for ILC2-derived LIF in CD45^+^ immune cell homing in response to PVM, we used Boolean-ILC2-Cre (BIC)-targeting mice^33^ intercrossed with *Lif*^flox/flox^ mice to produce ILC2^LIFKO^ mice (Extended Data Fig. 3k). Quantitative PCR (qPCR) confirmed the deletion of *Lif* in ILC2s, but not in T cells or B cells (Extended Data Fig. 3l). Following PVM infection of ILC2^LIFKO^ mice we again observed improved antiviral immunity, as indicated by reduced viral load (Extended Data Fig. 3m). Although lung eosinophils, neutrophils, alveolar macrophages and monocytes were unchanged (Extended Data Fig. 3n), there was a substantial impairment of CD45^+^ immune cell numbers in the MedLN (including T cells, B cells, pDCs and cDCs) but not in the lung (Fig. 2d and Extended Data Fig. 3o–s). This corresponded with reduced numbers of CCR7^+^CD45^+^ immune cells (Fig. 2g and Extended Data Fig. 4a–c), suggesting that the CCL21–CCR7 cell homing signal may be dysregulated across all these immune cell subsets in the absence of LIF. However, unlike LIFR^+^ pDCs which we showed can respond directly to LIF by upregulation of CCR7 expression, we did not observe changes in the proportions of other lung immune cells expressing CCR7 in the presence or absence of LIF signalling (Extended Data Fig. 4d). Nevertheless, the immune cell homing defect could be reversed by injection of PVM-infected LIF-cKO mice with rLIF (Fig. 2h and Extended Data Fig. 4e,f), indicating that the immune cell deficit is LIF dependent. These results raised the possibility that, rather than regulating only CCR7 expression on pDCs, LIF also controls the expression of CCR7 ligands, CCL21 and/or CCL19 to modulate all CCR7^+^CD45^+^ immune cell homing. These data suggest the existence of a previously unappreciated role for ILC2-derived LIF in regulation of immune cell egress from lung to MedLN.

## LIF induces CCL21 from lymphatic endothelial cells

**Extended Data Fig. 5:**
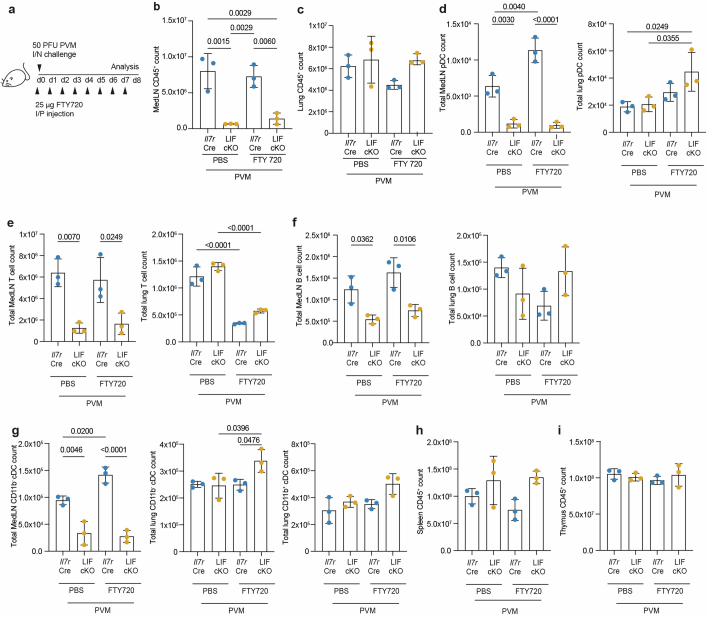
FTY720 treatment of conditional LIF-deficient mice. **a**, Schematic of PVM intranasal infection with PBS or FTY720 treatment. Flow cytometry analysis of **b**, MedLN CD45^+^ cells; **c**, lung CD45^+^ cells; **d**, MedLN and lung pDCs; **e**, MedLN and lung T cells; **f**, MedLN and lung B cells; **g**, MedLN and lung cDCs; **h**, spleen CD45^+^ cells; and **i**, thymus CD45^+^ cells in *Il7r*^*Cre*^ and LIF-cKO mice treated with PBS or FTY720 following PVM challenge. (n = 3). **b-i**, one-way ANOVA with Tukey’s multiple comparisons test. Data mean ± SEM. Data are representative of two (b-i) independent experiments with similar results. Source Data

For investigation of this LIF-regulated immune cell homing pathway, we first assessed whether immune cells were failing to be retained in the MedLN. LIF-cKO or control mice were infected with PVM and treated with either sphingosine 1-phosphate analogue FTY720 (which blocks lymphocyte egress from LNs^34^) or PBS (Extended Data Fig. 5a). No accumulation of lymphocytes was observed in either the LNs or lung of FTY720-treated LIF-cKO mice (Extended Data Fig. 5b–g), in contrast to PBS-treated controls, whereas there were comparable numbers of CD45^+^ immune cells in the spleen and thymus (Extended Data Fig. 5h,i), supporting the proposal that T cells, B cells and DCs were not reaching the LN in the absence of LIF.

**Fig. 3:**
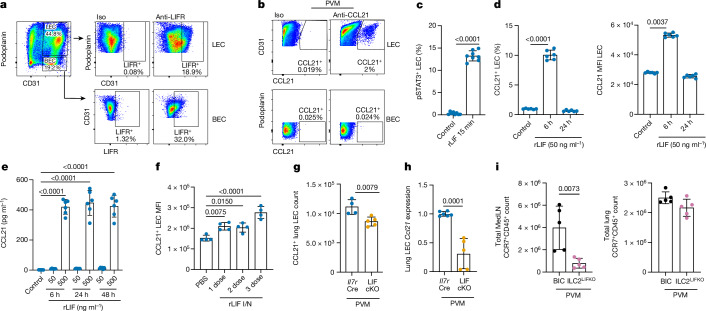
ILC2-derived LIF induces CCL21 production by LECs. **a**,**b**, Flow cytometry analysis of LIFR expression in lung LECs and BECs in naive mice (**a**) and CCL21 expression in lung LECs and BECs following PVM challenge (**b**). **c**,**d**, Flow cytometry analysis of phosphorylated STAT3^+^ in cultured LECs following rLIF treatment (*Il7r*^Cre^, *n* = 8; LIF-cKO, *n* = 8) (**c**) and CCL21^+^ LEC percentage and CCL21 median fluorescence intensity (MFI) following rLIF treatment (*Il7r*^Cre^, *n* = 6; LIF-cKO, *n* = 6) (**d**). **e**, ELISA of CCL21 concentration in in vitro cultured LEC-conditioned media following rLIF treatment (control, *n* = 5; rLIF, *n* = 6). **f**,**g**, Flow cytometry analysis of CCL21 MFI of lung LECs in WT mice following rLIF challenge at the indicated time points (*n* = 4) (**f**) and CCL21^+^ lung LEC numbers in *Il7r*^*Cre*^ and LIF-cKO mice following PVM challenge (*Il7r*^Cre^, *n* = 4; LIF-cKO, *n* = 5) (**g**). **h**, qPCR analysis of *Ccl21* relative expression in purified lung LECs from *Il7r*^*Cre*^ and LIF-cKO mice following PVM challenge (*Il7r*^Cre^, *n* = 6; LIF-cKO, *n* = 5). **i**, Flow cytometry analysis of MedLN and lung CCR7^+^CD45^+^ cell numbers in BIC and ILC2^LIFKO^ mice following PVM challenge (*n* = 5). **c**,**d**,**f**–**i**, Unpaired two-sided *t*-test; **e**, one-way ANOVA with Tukey’s multiple-comparisons test. Data presented as mean ± s.e.m. **a**–**d**,**f**–**i**, Data are representative of two independent experiments with similar results; **e**, experiments are pooled data from two independent experiments. Source Data

**Extended Data Fig. 6:**
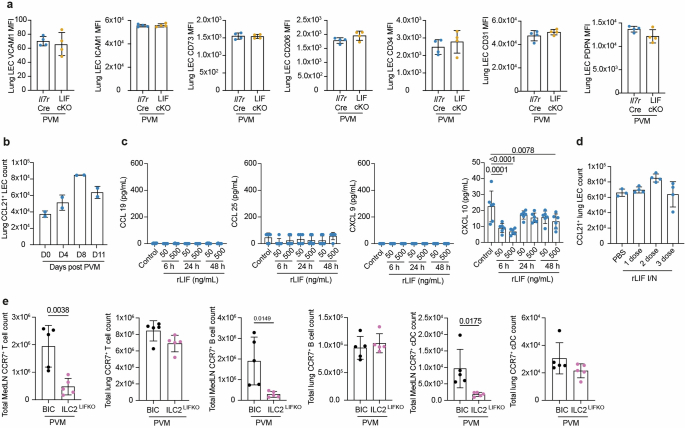
Adhesion molecule and chemokine receptor expression by lung LECs. Flow cytometry analysis of: **a**, lung LEC for adhesion molecules VCAM1, ICAM1, CD73, CD206, CD34, CD31, Podoplanin (PDPN) from *Il7r*^*Cre*^ and LIF-cKO mice following PVM challenge (n = 4), **b**, CCL21^+^ lung LEC numbers in wildtype mice following PVM challenge at indicated timepoints (n = 2). **c**, ELISA of CCL19, CCL25, CXCL9, CXCL10 concentration in in-vitro cultured LEC conditioned media following rLIF treatment for 6 h or 24 h or 48 h (n = 6). **d**, Flow cytometry analysis of CCL21^+^ lung LEC numbers in wildtype mice following rLIF challenge at indicated timepoint (PBS = 3, rLIF =4). **e**, Flow cytometry analysis of MedLN and lung CCR7^+^ T cells, CCR7^+^ B cells, and CCR7^+^ cDCs from BIC and ILC2^LIFKO^ mice following PVM challenge (n = 5). **a,d,e**, unpaired two-sided *t*-test, **c**, one-way ANOVA with Tukey’s multiple comparisons test. Data mean ± SEM. Data are representative of two (a, b, d, e) independent experiments with similar results. Experiments in (c) are pooled data from two independent experiments. Source Data

We next re-examined the identity of the endothelial LIFR^+^ cells that we had observed in the lung and identified them as CD45^−^CD31^+^podoplanin^+^ lymphatic endothelial cells (LECs), which comprise the wall of pulmonary lymphatic vessels, and blood vascular endothelial cells (BECs, CD45^−^CD31^+^podoplanin^−^), which line the blood vessels (Fig. 3a). Both LECs and BECs have roles in immune cell recruitment through their expression of cell adhesion molecules and chemokines^35,36^. Examination of cell adhesion molecules expressed by LECs failed to show differences between LIF-cKO and control mice (Extended Data Fig. 6a). However, lung LECs expressed CCL21 (the ligand for CCR7) whereas BECs did not (Fig. 3b), and CCL21 increased during PVM infection (Extended Data Fig. 6b). In vitro expanded LECs responded to LIF by phosphorylation of STAT3 (Fig. 3c) and rapid upregulation of intracellular CCL21 (Fig. 3d), resulting in a 400-fold increase in secreted CCL21 (Fig. 3e). By contrast, LEC expression of chemokines CCL19, CCL25, CXCL9 and CXCL10 was either not detectable or not induced by LIF stimulation (Extended Data Fig. 6c). Intranasal administration of LIF to WT mice increased LEC expression of CCL21 (Fig. 3f) although it did not increase the total numbers of CCL21^+^ LECs (Extended Data Fig. 6d). Notably, PVM-infected LIF-cKO mice had fewer CCL21-expressing LECs (Fig. 3g), which expressed fewer *Ccl21* transcripts than LEC from control mice (Fig. 3h). These data demonstrate that ILC2-derived LIF stimulates LECs to produce CCL21, a necessary cue for the efficient migration of CCR7^+^ immune cells to LNs^17,18,37^. Indeed, the defect in CCL21 expression was associated with a deficit in CCR7^+^ immune cells in the MedLN, but not lung, when LIF was deleted specifically in ILC2s (Fig. 3i and Extended Data Fig. 6e).

## ILC2–LIF axis helps protect from viral reinfection

**Fig. 4:**
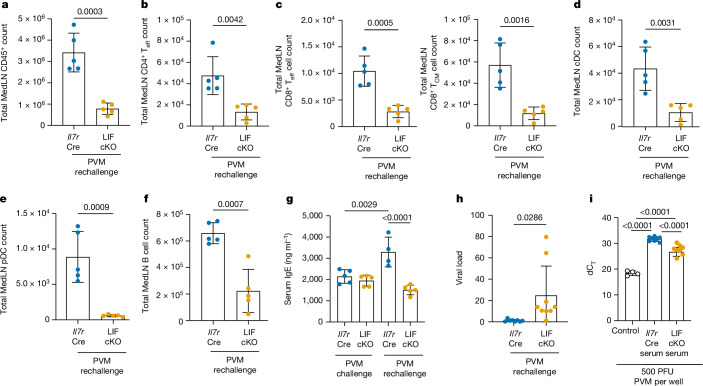
Defective immunity and MedLN colonization persist even following viral reinfection. **a**–**f**, Flow cytometry analysis of CD45^+^ (**a**), CD4^+ ^T_eff_ cell (**b**), CD8^+ ^T_eff_ cell and T_CM_ cell (**c**), cDC (**d**), pDC (**e**) and B cell numbers (**f**) in *Il7r*^Cre^ and LIF-cKO mice following PVM rechallenge (*n* = 5). **g**, ELISA of serum IgE in *Il7r*^Cre^ and LIF-cKO mice following PVM primary (*Il7r*^Cr^, *n* = 5; LIF-cKO, *n* = 5) and secondary (*Il7r*^Cre^, *n* = 4; LIF-cKO, *n* = 5) challenge. **h**, Lung viral load in *Il7r*^Cre^ and LIF-cKO mice following PVM rechallenge (*Il7r*^Cre^, *n* = 8; LIF-cKO, *n* = 9). **i**, In vitro viral neutralization assay with *Il7r*^Cre^ and LIF-cKO serum following PVM rechallenge. dC_T_ was calculated as C_T_(PVM)-C_T_ (hamster GAPDH) (control, *n* = 4; PVM rechallenged *Il7r*^Cre^ serum, *n* = 8; PVM rechallenged LIF-cKO serum, *n*  =8). **a**–**f,h**,**i**, Unpaired two-sided *t*-test; **g**, one-way ANOVA with Tukey’s multiple-comparisons test. Data presented as mean ± s.e.m. **a**–**g**,**i**, Data are representative of two independent experiments with similar results; **h**, experiments are pooled data from two independent experiments. PFU, plaque-forming units. Source Data

**Extended Data Fig. 7:**
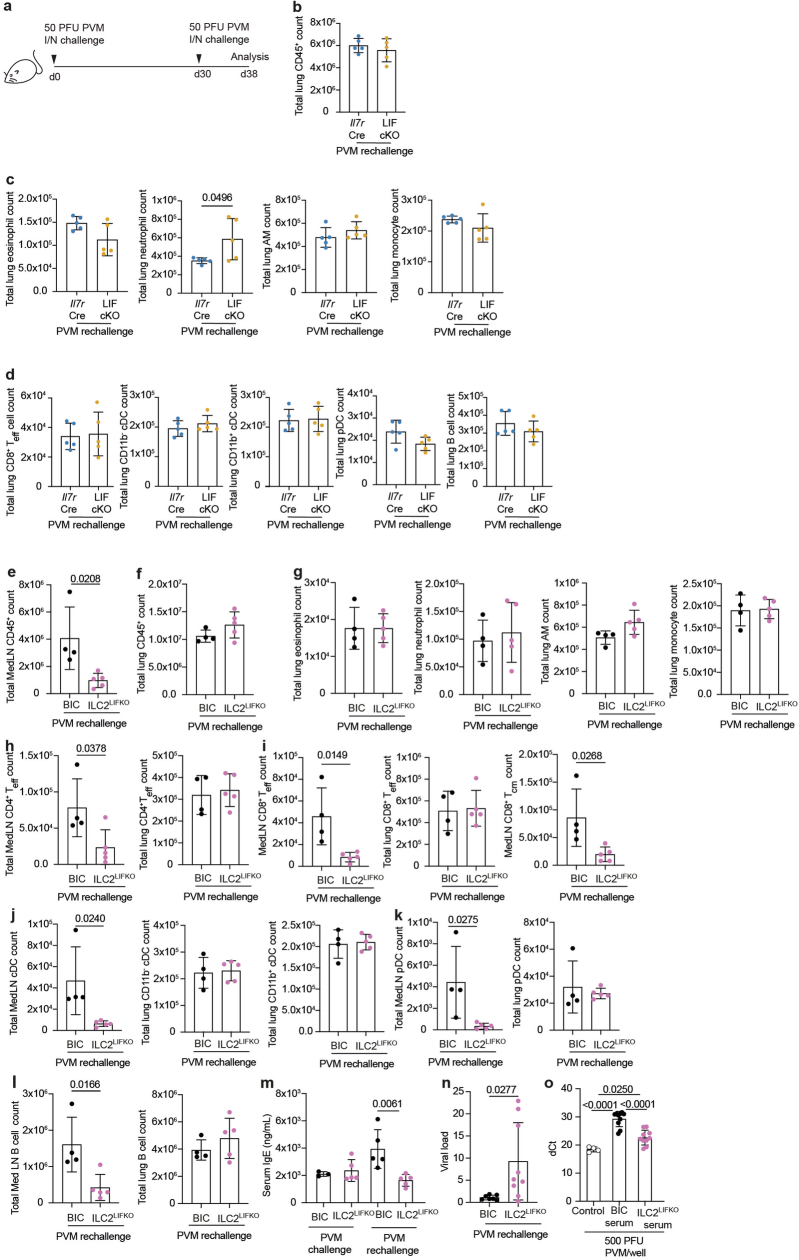
Pneumovirus reinfection in conditional LIF-deficient mice. **a**, Schematic of PVM intranasal primary and secondary infection. Flow cytometry analysis of lung: **b**, CD45^+^; **c**, eosinophil, neutrophil, alveolar macrophage (AM) and monocyte; **d**, CD4^+^T_Eff_ cells, CD8^+^T_Eff_ cells and T_CM_ cells, DCs, pDCs and B cell numbers from *Il7r*^Cre^ and LIF-cKO mice following PVM rechallenge (n = 5). Flow cytometry analysis of **e**, MedLN CD45^+^ cells; **f**, lung CD45^+^ cells; **g**, lung eosinophils, neutrophils, alveolar macrophages (AM) and monocytes; **h**, MedLN and lung CD4^+^ T_Eff_ cells; **i**, MedLN and lung CD8^+^ T_Eff_ and CD8^+^ T_CM_ cells; **j**, MedLN and lung cDCs; **k**, MedLN and lung pDCs, and **l**, MedLN and lung B cells in BIC and ILC2^LIFKO^ mice following PVM rechallenge (BIC = 4 and ILC2^LIFKO^ = 5). **m**, ELISA of serum IgE from BIC and ILC2^LIFKO^ mice following PVM primary and secondary challenge (PVM challenged BIC = 3, PVM challenged ILC2^LIFKO^ = 5, PVM rechallenged BIC = 5, PVM rechallenged ILC2^LIFKO^ = 5;). **n**, Lung viral load in BIC and ILC2^LIFKO^ mice following PVM rechallenge (BIC = 7 and ILC2^LIFKO^ = 9). **o**, In vitro viral neutralization assay with BIC and ILC2^LIFKO^ mice serum following PVM rechallenge. dC_T_ was calculated as C_T_(PVM)-C_T_(hamster GAPDH) (control = 4, PVM rechallenged BIC serum =10 and PVM rechallenged ILC2^LIFKO^ serum =10). **b-o**, unpaired two-sided *t*-test. Data mean ± SEM. Data are representative of two (b-m) independent experiments with similar results. Experiments in (n,o) are pooled data from two independent experiments. Source Data

We next determined whether the defective primary immune response of LIF-cKO mice to viral infection could be overcome by secondary reinfection 30 days following the original viral challenge (Extended Data Fig. 7a). Despite comparable numbers of immune cells in the lungs of both LIF-cKO and control mice (Extended Data Fig. 7b), there was a marked deficit in the cellularity and immune cell composition in MedLN of LIF-cKO mice (Fig. 4a–f). This included fewer CD4^+^ and CD8^+^ T effector (T_eff_, CD44^hi^CD62L^−^) cells, CD8^+^ T central memory (T_CM_, CD44^hi^CD62L^+^) cells, cDCs, pDCs and B cells (Fig. 4b–f and Extended Data Fig. 7c,d). B cell deficiency was reflected in a failure to upregulate the expression of circulating IgE (Fig. 4g), which is normally elevated in response to PVM infection^38,39^. Similarly, ILC2^LIFKO^ mice had fewer CD45^+^ immune cells in their MedLN following PVM rechallenge (Extended Data Fig. 7e–g), including T_eff_ cells, T_CM_ cells, cDCs, pDCs and B cells in the MedLN (Extended Data Fig. 7h–l). A deficit in B cells also correlated with decreased circulating serum IgE (Extended Data Fig. 7m). Furthermore, whereas the secondary immune response cleared the virus from the lungs of control mice, the deficit in adaptive immunity in both LIF-cKO mice (Fig. 4h) and ILC2^LIFKO^ mice (Extended Data Fig. 7n) correlated with persistent viral infection. This was associated with reduced viral neutralization activity in the serum of both LIF-cKO (Fig. 4i) and ILC2^LIFKO^ mice (Extended Data Fig. 7o) as compared with controls. These results indicate that the generation of acquired immunity to PVM is impaired in the absence of ILC2-derived LIF.

## LIF deficiency promotes allergen-induced iBALT

**Fig. 5:**
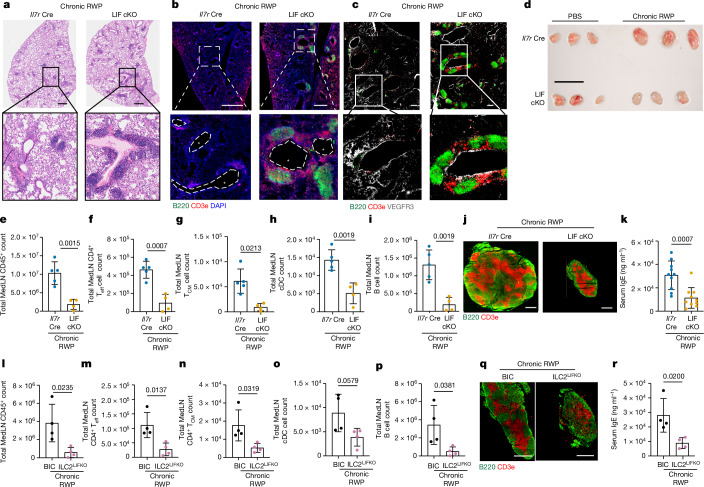
Chronic allergen challenge leads to marked iBALT accumulation in the absence of ILC2-derived LIF. **a**,**b**, Lung histology (**a**) and immunofluorescence (**b**) of *Il7r*^*Cre*^ and LIF-cKO mice following chronic RWP challenge. B cells were stained with B220 antibody (green), T cells with CD3e antibody (red) and nuclei with DAPI (blue). **c**, Immunofluorescence as in **b**, with B cells stained with B220 antibody (green), T cells with CD3e antibody (red) and lymphatic vessels with VEGFR3 (grey). **d**, Representative image of MedLN from *Il7r*^*Cre*^ and LIF-cKO mice following PBS or chronic RWP challenge. **e**–**i**, Flow cytometry analysis of numbers of CD45^+^ cells (**e**), CD4^+ ^T_eff_ cells (**f**), CD4^+^ T_CM_ cells (**g**), cDCs (**h**) and B cells (**i**) from *Il7r*^*Cre*^ and LIF-cKO mice following chronic RWP challenge (*Il7r*^Cre^, *n* = 5; LIF-cKO, *n* = 4). **j**, Immunofluorescence of MedLN from *Il7r*^*Cre*^ and LIF-cKO mice following chronic RWP challenge. B cells were stained with B220 antibody (green) and T cells with CD3e antibody (red). **k**, ELISA of serum IgE from *Il7r*^Cre^ and LIF-cKO mice following chronic RWP challenge (*n* = 10). **l**–**p**, Flow cytometry analysis of CD45^+^ cells (**l**), CD4^+ ^T_eff_ cells (**m**), CD4^+^ T_CM_ cells (**n**), cDCs (**o**) and B cells (**p**) from BIC and ILC2^LIFKO^ mice following chronic RWP challenge (*n* = 4). **q**, Immunofluorescence of MedLN from BIC and ILC2^LIFKO^ mice following chronic RWP challenge. B cells were stained with B220 antibody (green) and T cells with CD3e antibody (red). **r**, ELISA of serum IgE from BIC and ILC2^LIFKO^ mice following chronic RWP challenge (*n* = 4). **e**–**i**,**k**–**p**,**r**, Unpaired two-sided *t*-test. Data presented as mean ± s.e.m. **a**–**j**,**l**–**r**, Data are representative of two independent experiments with similar results; **k**, experiments are pooled data from two independent experiments. Scale bars, 10 µm (**a**), 70 µm (**b**), 45 µm (**c**), 10 mm (**d**), 50 µm (**j**), 70 µm (**q**). Source Data

**Extended Data Fig. 8:**
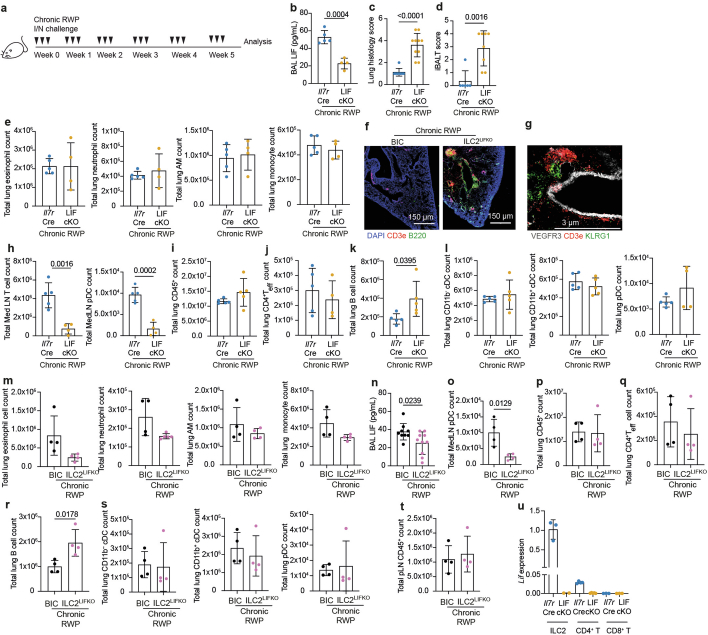
Chronic RWP challenge of conditional LIF-deficient mice. **a**, Schematic of chronic RWP intranasal challenge protocol. **b**, ELISA of LIF in BAL from *Il7r*^*Cre*^ and LIF-cKO mice following chronic RWP challenge (*Il7r*^Cre^ = 5, LIF-cKO =4). **c**, Lung histology scores from *Il7r*^*Cre*^ and LIF-cKO mice following chronic RWP challenge (*Il7r*^Cre^ = 8, LIF-cKO =10). **d**, Lung iBALT scores from *Il7r*^*Cre*^ and LIF-cKO mice following chronic RWP challenge. (*Il7r*^Cre^ = 6, LIF-cKO =8). **e**, Flow cytometry analysis of lung eosinophils, neutrophils, alveolar macrophages and monocytes in *Il7r*^*Cre*^ and LIF-cKO mice following chronic RWP challenge (*Il7r*^Cre^ = 5, LIF-cKO =4). **f**, Immunofluorescence histology of lung showing tertiary lymphoid tissue formation in BIC and ILC2^LIFKO^ mice following chronic RWP challenge, B cells stained with B220 antibody (green), T cells with CD3e antibody (red) and nuclei with DAPI (blue). **g**, Immunofluorescence histology of lung from LIF-cKO mouse after chronic RWP intranasal challenge. KLRG1 (green), CD3e (red) and VEGFR3 (grey). Flow cytometry analysis of **h**, MedLN T cells and pDCs (*Il7r*^Cre^ = 5, LIF-cKO =4); **i**, lung CD45^+^ cells (*Il7r*^Cre^ = 5, LIF-cKO =5), **j**, lung CD4^+^ T_Eff_ cells (*Il7r*^Cre^ = 5, LIF-cKO =4), **k**, lung B cells (*Il7r*^Cre^ = 5, LIF-cKO =5), **I**, lung cDCs (*Il7r*^Cre^ = 5, LIF-cKO =5) and pDCs (*Il7r*^Cre^ = 5, LIF-cKO =4) from *Il7r*^*Cre*^ and LIF-cKO mice following chronic RWP challenge. **m**, Flow cytometry analysis of lung eosinophils, neutrophils, alveolar macrophages (AM) and monocytes in Boolean Cre (BIC) and BIC x *Lif*^flox/flox^ (ILC2^LIFKO^) mice following chronic RWP challenge (n = 4). **n**, ELISA of LIF in BAL from BIC and ILC2^LIFKO^ mice following chronic RWP challenge (BIC = 9, ILC2^LIFKO^ = 10). Flow cytometry analysis of **o**, MedLN pDCs; **p**, lung CD45^+^ cells; **q**, lung CD4^+^ T_Eff_ cells; **r**, lung B cells; **s**, lung cDCs and pDCs, and **t**, inguinal lymph node (pLN) CD45^+^ cells in BIC and ILC2^LIFKO^ mice following chronic RWP challenge (n = 4). **u**, qPCR analysis of *Lif* expression in lung ILC2, CD4^+^ and CD8^+^ T cell purified from *Il7r*^*Cre*^ and LIF-cKO mice following chronic RWP challenge (n = 2-4). **b-e, h-t** unpaired two-sided *t*-test. Data mean ± SEM. Data are representative of two (b, e, f, g, h, i, j, k, l, m, o, p, q, r, s, t, u) independent experiments with similar results. Experiments in (c, d, n) are pooled data from two independent experiments. Source Data

Finally we wondered how the LIF-dependent defect in immune cell homing would impact more chronic models of inflammatory disease, such as persistent type 2 allergen-induced inflammation used to model allergy and asthma^40^. Consequently we challenged LIF-cKO mice intranasally with RWP for 5 consecutive weeks (Extended Data Fig. 8a). Even following such protracted immune challenge, the levels of LIF were reduced in the lungs of LIF-cKO mice (Extended Data Fig. 8b). Histological analysis of lung from RWP-challenged LIF-cKO mice showed the formation of iBALT (Fig. 5a–c and Extended Data Fig. 8c,d), although lung eosinophils, neutrophils, alveolar macrophages and monocytes were unchanged (Extended Data Fig. 8e). iBALT associates with localized immune responses^15,41^. Fluorescent imaging confirmed T cells and B cells organized in regions rich in CD3^−^KLRG1^+^ ILC2s and close to pulmonary lymphatic vessels, labelled with VEGFR3, in both LIF-cKO and ILC2^LIFKO^ mice (Fig. 5c and Extended Data Fig. 8f,g).

Notably, even after such sustained RWP antigen challenge, MedLNs were smaller in LIF-cKO mice due to a deficit in CD45^+^ immune cells (Fig. 5d,e), including CD4^+^ T_eff_ cells, CD4^+^ T_CM_ cells, DCs and B cells in MedLN (but not in lung) as compared with controls (Fig. 5f–i and Extended Data Fig. 8h–l). Immunofluorescence staining of MedLN confirmed the difference in MedLN size (Fig. 5j), which suggested a potential impairment of adaptive immunity. Indeed, chronic RWP challenge of LIF-cKO mice led to considerably reduced circulating IgE compared with controls (Fig. 5k). In ILC2^LIFKO^ mice, lung eosinophils, neutrophils, alveolar macrophages and monocytes were unchanged (Extended Data Fig. 8m) but they showed reduced BAL LIF levels (Extended Data Fig. 8n) and fewer CD45^+^ immune cells in their MedLN following chronic RWP challenge (Fig. 5l). A deficit in T_eff_ cells, T_CM_ cells, DCs and B cells in MedLN (Fig. 5m–q and Extended Data Fig. 8o), but not in the lungs (Extended Data Fig. 8p–s), correlated with a decrease in serum IgE (Fig. 5r). The inguinal LN, which does not drain the lung, showed no changes in cellularity following lung challenge, indicating that the effects of LIF deletion do not impact LNs not directly involved in the antigen-driven immune reaction (Extended Data Fig. 8t). These findings are in line with ILC2s continuing to be an indispensable source of LIF even following 5 weeks of antigen challenge (Extended Data Fig. 8u), although it is also possible that T cells may start to contribute as the response progresses. Finally, it is notable that the formation of iBALT in lung tissue did not rescue the deficit in total circulating serum IgE levels in either LIF-cKO or ILC2^LIFKO^ mice (Fig. 5r). Thus, responses to allergen in the absence of ILC2-derived LIF become amplified in the lung, leading to inappropriate tissue-localized iBALT reactions similar to those in asthma and allergy, but also to impaired systemic responses.

## Discussion

**Extended Data Fig. 9:**
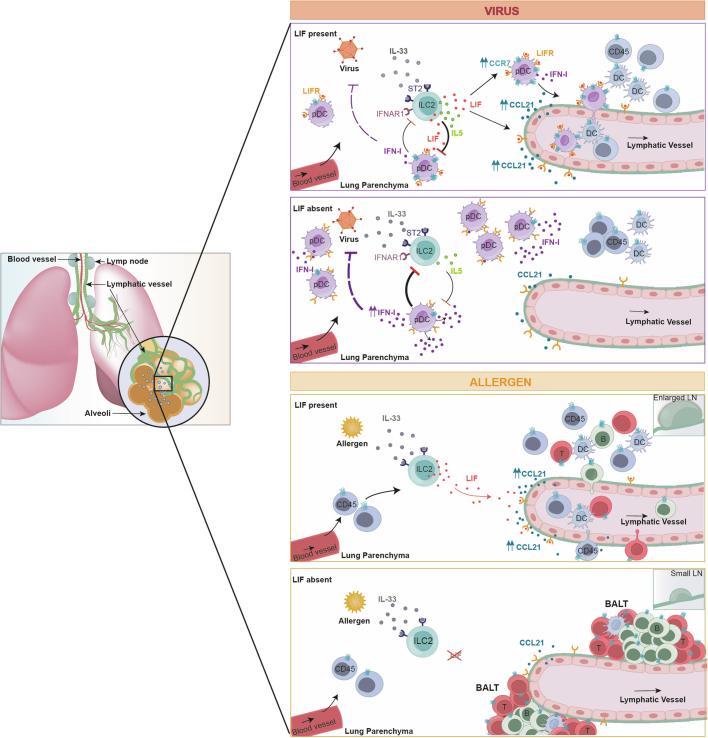
Schematic of the roles of ILC2-derived LIF regulation of immune cell migration during pulmonary responses to viral infection and allergen challenge. Source Data

Here we identified that LIF, produced by ILC2s, is essential for ensuring the efficient transition from lung tissue-localized immune reactions to LN-mediated systemic immunity. In the absence of ILC2-derived LIF, both antiviral and allergen-induced respiratory immune responses remained lung-centric with minimal immune cell seeding and expansion within draining LNs. This resulted in enhanced antiviral immunity but abnormal formation of iBALT in response to allergen challenge. We determined that LIF can both rapidly and directly promote CCR7 expression on pDCs and potently induce CCL21 chemokine production from LIFR^+^ LECs to enhance CCR7^+^ immune cell migration (including DCs and T cells) to the LNs to prime adaptive immunity (Extended Data Fig. 9). Indeed, conditional LIF deficiency closely phenocopies disruption of the CCR7–CCL21 chemokine pathway^15–18^, which is critical for the trafficking of antigen-loaded DCs to LNs via the afferent lymphatics where they stimulate antigen-specific T cells to promote systemic immunity^17^. As a result, mice deficient in CCR7, CCL21 and CCL19, or following lung-specific ablation of CCL21-producing LECs, develop anomalous iBALT^15,17,18^.

In the lungs, ILC2s and other lymphocytes commonly localize in the adventitial ‘cuffs’ within stromal niches close to lymphatic and blood vessels^28,42^. Here ILC2s sense stromal cell products or factors present in the fluid draining from the alveolar parenchyma to the lymphatics. These spatially restricted microenvironments leave ILC2s well placed to promote the local CCL21 chemokine gradients required to guide immune cell egress to the afferent lymphatics for transit to draining LNs^43,44^. Retention of DCs in the tissue would prolong their exposure to ILC2-derived cytokines such as GM-CSF, IL-4 and IL-13 (refs. ^45,46^), thereby locally activating DCs and promoting tissue-localized T cell responses^45–47^. Indeed, ILC2-derived IL-13 can stimulate DCs to produce the chemokine CCL17, which promotes the attraction of T_H_2 cells^48^. In the absence of ILC2-produced LIF we did not observe excessive accumulation of immune cells in the lungs following viral or allergen challenge, presumably because fewer activated immune cells are generated in the LNs for release back into the blood circulation to traffic back to the lungs. Thus, inefficient cDC migration and tissue-localized T cell priming would promote dysregulated tertiary lymphoid structures to the detriment of normally coordinated systemic immunity in draining LNs.

iBALT is beneficial for focusing tissue-localized immune reactions against viral infection^17,18,37^. Although we did not find archetypal iBALT in the lungs of conditional LIF-deficient mice following PVM infection (which does not form chronic infections^38,39^), we observed increased cell aggregates and retention of pDCs in the lung. This correlated with increased type I IFN, improved viral clearance and a reduced type 2 response often associated with tissue repair. Indeed, LIF is required to protect the lungs of mice infected with RSV or challenged with *Escherichia coli*-induced pneumonia^49,50^. It will be interesting to determine whether ILC2-derived LIF also has roles during bacterial infections in which pathogen-associated molecular pattern-driven immune activation is important. Because type I IFN can inhibit ILC2 functions^9,10^ and LIF can suppress pDC production of type I IFN^25^, this feedback mechanism may help to regulate the balance between antiviral type 1 immunity and reparative type 2 immunity to maintain, protect and restore lung health^9^. Indeed, dysregulated lung repair following primary infection may have contributed to the more persistent PVM virus infection we observed in response to PVM reinfection. Furthermore, ILC2-derived LIF was critical for establishing protective adaptive immunity to secondary infection with PVM virus, demonstrating the importance of the LIF signal in regulation of innate and acquired immunity.

iBALT formation also occurs with chronic allergen exposure, in which it is associated with detrimental lung inflammation^51^. Chronic allergen challenge of conditional LIF-deficient mice led to a pronounced and inappropriate accumulation of inflammatory cells in the lungs to form iBALT, to the detriment of draining LN responses. This failure of immune cells to move into the lymphatic system resulted in dysregulation of circulating IgE, an isotype indicative of atopic allergy and asthma-like responses^52^. This may be due directly to either impaired B cell migration or a shortfall in cDCs and T cells in the MedLN. Notably, mutations in the LIF receptor gene are associated with asthma in a population of Hutterites^53^. Although the authors did not investigate the mechanistic role of LIFR in asthma, the result raises the possibility that dysregulated LIFR signalling could contribute to abnormal immune cell homing in these individuals and contribute to symptoms. Also of note, RSV and influenza infection can exacerbate type 2-driven allergic asthma^14^, highlighting the finely balanced regulation required in the lungs to avoid detrimental inflammatory responses.

Our results support a key role for ILC2-derived LIF in controlling immune cell migration from the lung to the MedLN following immune stimulation. This is, at least in part, due to potent regulation by LIF of the CCL21–CCR7 pathway, leading to phenotypes that closely resemble deletion of components of this chemokine axis. However, it remains possible that additional LIF-dependent signals are contributory and yet to be discovered. Nevertheless, our finding that ILC2-derived LIF is a previously unappreciated regulator of immune cell trafficking raises fundamental questions about the role of this cytokine in human allergic disease and infections, but also in a broad range of other diseases—for example, inflammatory bowel disease and cancer in which intratumoural TLS^54^ correlate with low LIF expression and may represent a positive prognostic indicator for certain cancers^55^.

## Methods

### Mice

All mice were maintained in the Medical Research Council ARES animal facility under specific-pathogen-free conditions at 19–23 °C and 45–65% humidity with a 12/12 h light/dark cycle. In individual experiments, mice were matched for age, sex and background strain. All experiments undertaken in this study were performed with the approval of the LMB Animal Welfare and Ethical Review Body and the UK Home Office. C57BL/6 JOla controls were bred in house. Mouse strains *Il*7*r*^Cre^ (ref. ^56^), *Rora*^flox/flox^ (ref. ^57^)*, Il1rl1*^−/−^ (ref. ^58^), *Rag2*^−/−^, *Rag2*^−/−^*Il2rgc*^−/−^ (*Rag2*^−/−^*gc*^−/−^), *Lif* ^flox/flox^, BIC^33^, *Siglech*^Cre^ (ref. ^59^) and *Lifr*^–/flox^ were either on the C57BL/6J Ola background or back-crossed for at least six generations.

### Generation of *Lif*^flox/flox^ mice

To produce a *Lif* allele that could be conditionally deleted by Cre recombinase, we generated a homology-directed repair-template construct for use in combination with CRISPR–Cas9 to insert LoxP sites 5′ and 3′ of the final exon of both protein-coding annotated *Lif* transcripts (ENSMUSE00000656154). In addition, the construct included a neomycin selection cassette flanked by Frt sites to permit Flp-mediated excision, and both LoxP sites were followed by a BglII site to facilitate screening and verification of appropriately targeted embryonic stem cell clones (Extended Data Fig. 1r). Embryonic stem cells were transfected with this repair-template construct along with expression constructs for WT Cas9 and four single-guide RNAs, two targeting sequences 5′ and two targeting 3′ of the final *Lif* exon. Neomycin-resistant clones were screened for correct targeting initially by PCR and digested with BglII using 5′ primer pairs P1 and P2 such that a product cleaved by BglII indicated correct targeting. Clones were further verified by Southern blot analysis using 5′ and 3′ probes that both detected a 16.4 kb fragment in the WT allele and 4.9 and 7.6 kb fragments, respectively, in the targeted allele (Extended Data Fig. 1s). Guide RNA target sequences were: G1 (F) TAATGATTCTAGTTGCCTACAGG; G2 (F) TGGAGTCCCCATGTCACAGGTGG; G3 (F) TTCCTCCATCGGTCCAGGAGGGG; G4 (R) TACCCCTCCTGGACCGATGGAGG. Screening primers were: P1 TAGGAAGCCAGAGTCTAGTGGCAGTTTTAAGAGATGG; P2 AAGGCTTCTTTGTCAGAGTGGTCGG. Primers for generation of probes were: 5′ probe 1 fwd CCTGCCACCCCCTTAACCTCCATAAGTGAAAAGCAAGTGG; 5′ probe 1 rev. ACTGGGCCTGCTAGGGGTTTGACAG; 3′ probe 1 fwd TGATGGAGCTGTGGGATGGG; 3′ probe 1 rev. ACACACTCGGGCTCCATTATGC.

### Generation of LIFR conditional mice

For generation of pDC-specific LIFR knockout (*SiglecH*^Cre/Cre^ *Lifr*^−/flox^) mice, pDC^Cre^ (*SiglecH*^Cre/Cre^) mice were crossed with *Lifr*^−/flox^ to delete exon 5 of *Lifr* (transcript variant 1). Both Tg(Siglech-Cre,-mCherry)^59^ and Lifr^tm1a(EUCOMM)Hmgu^ (ref. ^25^) mice were obtained from the European mouse mutant archive. To delete the lacZ–neomycin-resistance cassette and generate mice with a *lox*P-flanked *Lifr* allele (EuComm *Lifr*^*tm1c*^ is denoted as *Lifr*^flox^ in this paper), chimaeras were bred to FLPe C57BL/6 mice. However, we also detected inefficient *Lifr*^flox^ allele recombination and consequently analysed pDC^Cre^ × *Lifr*^−/flox^ mice in which two Cre-mediated recombination events occurred to produce LIFR deficiency, with pDC^Cre^ × *Lifr*^−/+^ mice as controls. Despite two Cre alleles and one functional flox allele, pDC^Cre^ × *Lifr*^−/flox^ mice showed a reduction in LIFR expression of only 50% in pDCs (Extended Data Fig. 1t).

### Mouse challenge protocols

#### IL-33-induced type 2 lung inflammation

Mice were challenged intranasally with IL-33 (Biolegend; 0.25 µg in 40 µl of PBS) on 3 consecutive days. All tissues were harvested 24 h following the final dose.

#### Neutralizing LIF antibody treatment

Mice were intranasally challenged with IL-33 (0.25 µg in 40 µl of PBS) and intraperitoneally injected with 100 µg of either isotype antibody (R&D systems) or anti-LIF neutralizing antibody (R&D systems) on 3 consecutive days. All tissues were harvested 24 h following the final dose.

#### rLIF intranasal challenge

Mice were intranasally challenged with rLIF (R&D systems; 1 µg in 40 µl of PBS) on 3 consecutive days. All tissues were harvested 24 h following the final dose. For the pDC migration kinetics experiment, mice were intranasally challenged with one rLIF dose (1 µg in 40 µl of PBS) and tissues harvested at 6 or 24 h after challenge.

#### RWP-induced type 2 lung inflammation

Mice were intranasally challenged with RWP (300 μg of protein per dose, *Ambrosia artemisiifolia*, short form; Greer Laboratories) on 3 consecutive days. All tissues were harvested 24 h following the final dose. For the chronic lung inflammation model, mice were intranasally challenged with RWP thrice weekly over 5 weeks. All tissues were harvested on day 38.

#### FITC-dextran-labelled cell migration

Mice were challenged intranasally with IL-33 (Biolegend, 0.25 µg) and 40 kDa FITC-dextran (Sigma-Aldrich, 40 µg in 50 µl of PBS) on 3 consecutive days. All tissues were harvested 24 h following the final dosing.

### Mouse infection models

#### PVM infection

Mice were infected intranasally with a single dose of PVM (50 PFU in PBS). PVM strain J3666 stock was a gift from A. J. Easton. All tissues were harvested at 8 days postinfection unless stated otherwise. For the CCL21 kinetics experiment, mice were intranasally challenged with PVM (50 PFU in PBS) and tissues harvested on 0, 4, 8 and 11 days postinfection.

For PVM rechallenge, mice were challenged with PVM (50 PFU in PBS) on days 0 and 30 and all tissues were harvested on day 38.

#### FITC-dextran-labelled cell migration

Mice were challenged intranasally with PVM (50 PFU) and 40 kDa FITC-dextran (Sigma-Aldrich, 40 μg in 50 µl of PBS). All tissues were harvested 3 days postinfection.

#### rLIF and PVM challenge

Mice were infected with a single dose of PVM (50 PFU in PBS) intranasally and treated with a daily dose of intranasal rLIF(1 μg) or PBS. All tissues were harvested on day 8 postinfection.

Mice were infected with a single dose of PVM (50 PFU in PBS) intranasally and, on day 8, postinfection intravenous CD45 labelling was performed by injection of 3 μg of anti-CD45 antibody (in 200 μl of PBS) via the tail vein. Mice were then culled 3 min after injection and tissues harvested.

#### FTY720 and PVM challenge

Mice were infected with a single dose of PVM (50 PFU in PBS) intranasally and injected intraperitoneally daily with either FTY720 (ref. ^34^) (25 μg in 250 µl; Enzo Life Sciences) or PBS. All tissues were harvested on day 8 postinfection.

### Tissue processing

#### BAL isolation

Mice were culled at the experimental endpoint, tracheae were exposed and BAL was performed by flushing the lungs three times with 0.5 ml of PBS. The fluid obtained was centrifuged at 350*g* for 5 min; supernatants were stored at −20 °C for cytokine detection.

#### Serum isolation

Mice were culled at the experimental endpoint and whole blood was collected. Blood samples were allowed to clot for 2 h at room temperature. Samples were centrifuged at 2,000*g* for 10 min and serum was collected and stored at −20 °C. For immunoglobulin enzyme-linked immunosorbent assay (ELISA), serum was diluted 1/50.

#### Viral load

Mice were culled at the experimental endpoint, and one lung lobe was snap-frozen in trizol (Invitrogen) and stored at −80 °C for RNA purification.

#### Tissue preparation

Lung tissue was predigested with 750 U ml^−1^ collagenase I (Gibco) and 0.3 mg ml^−^^1^ DNase I (Sigma-Aldrich) before obtaining a single-cell suspension at 37 °C for 30 min; tissue was then passed through a 70 μm cell strainer. For lymphocyte enrichment, a single-cell lung suspension was centrifuged through 30% Percoll (GE Healthcare) at 800*g* for 15 min. Spleen, thymus and mediastinal LN single-cell suspensions were prepared by passing tissue through a 70 μm cell strainer and lysing red blood cells. Single-bone marrow cell suspensions were prepared by flushing the femur and tibia with endotoxin-free PBS and lysing red blood cells.

### Flow cytometry

Single-cell suspensions were incubated with fluorochrome- or biotin-conjugated antibodies in the presence of anti-CD16/CD32 antibody (Fc block, clone 2.4G2), followed by fluorochrome-conjugated streptavidin where necessary. All samples were costained with a cell viability dye (Fixable dye eFluor780, Invitrogen) and analysed on either a 5-5-laser LSRFortessa system (BD Biosciences, BD FACSDiva software v.6.2) or spectral cytometer ID7000 (Sony Biotechnology). Either FACSAria Fusion systems or iCyt Synergy (70 μm nozzle, Sony Biotechnology) was used for cell sorting. Precision Count Beads (BioLegend) were used to calculate cell numbers. Intracellular transcription factor staining was performed using the Foxp3 staining kit (eBioscience) according to the manufacturer’s instructions. For lymphocyte intracellular cytokine staining, cells were cultured with complete RPMI supplemented with Cell Stimulation Cocktail or protein transport inhibitors (eBioscience) for 4 h at 37 °C. Intracellular cytokine staining was performed using BD Cytofix/Cytoperm Plus reagents (BD Biosciences) following the manufacturer’s instructions. The expression of LEC CCL21 was detected by additional staining with goat anti-mouse CCL21 (R&D systems) and anti-goat-Alexa 488 (Invitrogen), with no stimulation, and using BD Cytofix/Cytoperm Plus reagents (BD Biosciences) following the manufacturer’s instructions.

For intracellular phospho-STAT3 staining, cells were fixed with 2% paraformaldehyde (PFA) for 15 min and overnight permeabilization with 90% methanol at −20 °C, followed by incubation with fluorochrome antibodies diluted in 2% bovine serum albumin PBS.

Flow cytometric analysis, including unsupervised dimensionality reduction and clustering, was performed using FlowJo, LLC v.10 (BD) and associated plug-ins. Unless otherwise stated, pDCs are defined as LiveCD45^+^CD11b^−^F4/80^−^CD317^+^SiglecH^+^. Myeloid cells include cDCs and CD11b^−^ cDCs as LiveCD45^+^CD11b^−^CD11c^high^SiglecF^−^MHCII^high^; CD11b^+^ cDCs as LiveCD45^+^CD11c^−/intm^ SiglecF^−^Ly6G^−^CD19^−^TCRb^−^MHCII^high^; monocytes as LiveCD45^+^CD11b^+^ SiglecF^−^Ly6G^−^F4/80^+^MHCII^−^; and alveolar macrophages as CD45^+^CD11c^+^F4/80^+^SiglecF^+^. Eosinophils are defined as CD45^+^CD11c^−^F4/80^−^CD11b^+^Gr1^int^ SiglecF^+^; and neutrophils as CD45^+^CD11c^−^F4/80^−^CD11b^+^Ly6G^high^SiglecF^−^. T cells are defined as LiveCD45^+^TCRb^+^ and B cells as LiveCD45^+^CD19^+^TCRb^−^; CD4^+^ T cells as CD45^+^CD3^+^CD4^+^ and ILC2s as CD45^+^Lin^−^(CD3,CD4,CD8,CD19,CD11b,CD11c,FcεR1) CD127^+^ICOS^+^. Endothelial cells are defined as LiveCD45^−^CD31^+^, LECs as LiveCD45^−^CD31^+^PDPN^+^ and BECs as LiveCD45^−^CD31^+^ PDPN^−^.

All flow cytometry data were processed and analysed using FlowJo v.10, RRID: https://scicrunch.org/resolver/SCR_008520.

### In vitro cultured cells

#### Lung immune cell sorting

Mouse ILC2s were purified from IL-33-treated lungs (see Methods for IL-33-induced type 2 lung inflammation) as LiveCD45^+^Lineage^−^IL-7Rα^+^ST2^+^KLRG1^+^; and pDCs were purified from IL-33-treated lungs (see Methods for IL-33-induced type 2 lung inflammation) as LiveCD45^+^F4/80^−^CD11b^−^CD317^+^SiglecH. Cells were snap-frozen in trizol for RNA purification, and conditioned medium was collected and stored at −20 °C.

Mouse lung ILC2s and T cells for qPCR analysis were purified from PVM-challenged mice using the same gating strategy as for ILC2s: LiveCD45^+^Lineage^−^IL-7Rα^+^ST2^+^KLRG1^+^; CD4^+^ T cells as LiveCD45^+^TCRb^+^CD4^+^; CD8^+^ T cells as LiveCD45^+^TCRb^+^CD8^+^; BECs as LiveCD45^−^CD31^+^PDPN^−^; and LECs as LiveCD45^−^CD31^+^PDPN^+^. Purified cells were snap-frozen in trizol for RNA purification.

#### ILC2 in vitro stimulation

Purified ILC2s were cultured for 24 h with IL-7 (10 ng ml^−1^) and IL-2 (50 ng ml^−1^) with or without IL-33 (10 ng ml^−1^). Cells for RNA purification and conditioned media were collected and stored at −20 °C.

#### pDC culture, purification and activation

Bone marrow cells were obtained by flushing femurs and tibias with RPMI, followed by incubation with red blood cell lysis buffer for 5 min. Following washing, cells were cultured with RPMI containing 10% fetal calf serum, 1% penicillin/streptomycin, sodium pyruvate, non-essential amino acids, l-glutamine, β-mercaptoethanol and Flt3L (10 ng ml^−1^) for 7–10 days. Medium was refreshed on days 3 and 6. pDCs were sorted from bone marrow-derived cultures by fluorescent activated cell sorting as LiveCD45^+^CD11c^int^SiglecH^+^CD317^+^ cells. Purified pDCs were activated with CpG (6 μg ml^−1^, Invivogen) and treated with or without rLIF (500 ng ml^−1^) for 24 h.

#### Chemotaxis assay

Migration assays were performed using Millicell cell culture inserts (Merck Millipore) with 2–3 × 10^5^ cells per well. Purified pDCs were activated with CpG (6 μg ml^−1^) and treated with or without rLIF (10 ng ml^−1^) for 24 h. Activated pDCs were placed in inserts with 5 μm pores for 3 h in the presence or absence of cytokine rLIF (500 or 1000 ng ml^−1^) or chemokine rCCL21 (R&D systems) at 150 ng ml^−1^. The number of migrating cells was then evaluated using a flow cytometer. The results are expressed as migration index (number of migrating cells in chemokine/number of migrating cells in medium).

### Lung LEC purification and culture

The preparation of single-cell suspensions from lung tissues is described in ‘Tissue preparation’. CD31^+^ lung cells were isolated from lung cell suspension using magnetic beads (CD31 biotin, Streptavidin dynabeads)^60^. Isolated CD31^+^ lung cells were seeded onto 0.2% gelatin-coated, six-well plates and cultured on complete growth medium. consisting of ECGS (Corning), 20% fetal bovine serum, 1% penicillin/streptomycin, sodium pyruvate, non-essential amino acids and 25 mM HEPES, in a humidified incubator with a gas mixture of 21% O_2_ and 5% CO_2_ at 37 °C until 70–80% confluence was achieved (usually reached in 4–7 days). Endothelial cells were detached with Accutase (Stemcell Technologies) and purified using magnetic beads (podoplanin biotin, Streptavidin dynabeads). Purified LECs were seeded onto 0.2% gelatin-coated, six-well plates, cultured in complete growth medium and used for experiments.

### In vitro LEC treatment

Isolated LECs were treated with or without rLIF for 6 or 24 h, detached using Accutase and stained for flow cytometry analysis.

### ELISA and MAGPIX Luminex Array

Culture supernatant was collected and stored at −20 °C until analysis. Serum IgE was measured by ELISA (Invitrogen). LIF, IL-5, type I IFN, CCL19, CCL21, CCL25, CXCL9 and CXCL10 were measured using ProcartaPlex kits (Invitrogen).

### Virus neutralization assay

Sera from PVM-rechallenged mice were diluted in a 1:10 ratio and heat-inactivated at 55 °C for 30 min. An equal volume of PVM at 500 PFU per well concentration (1:20 final serum dilution) was incubated with serum for 1 h at 37 °C and 5% CO_2_. BHK-21 cell monolayers (1 × 10^5^ per well) were infected with the virus mixture and incubated for 72 h at 37 °C and 5% CO_2_. Before harvesting the cells were washed three times with PBS, snap-frozen in Trizol (Invitrogen) and stored at −80 °C for RNA purification.

### qPCR with reverse transcription

RNA was purified using Direct-zol RNA Purification Kits. For assessment of viral load, frozen tissue samples were homogenized before RNA purification. Complementary DNA synthesis was performed using SuperScript IV Reverse Transcriptase and oligo d(T)_20_ (Invitrogen). PVM viral load was tested with forward primer 5′-GCCTGCATCAACACAGTGTGT and reverse primer 5′-GCCTGATGTGGCAGTGCTT^38^ in a SYBR green qPCR assay. For lung samples, the mouse HPRT gene was used as an internal control. For the PVM neutralization assay, the dC_T_ for viral amplification was measured with respect to the hamster GAPDH gene. For other qPCR analyses, commercially available Taqman gene expression assays (Applied Biosystems; Extended Data Table 1) were used. Samples were run on the ViiA7 real-time PCR system (Applied Biosystems).

### RNA-seq

Cells were sorted by flow cytometry into PBS and 50% fetal calf serum. and RNA was extracted using the RNeasy Plus Micro kit (Qiagen). Following assessment using a Bioanalyser (Agilent), RNA was processed for RNA-seq using Ovation RNA-seq System v.2 (Nugen), fragmented by a Covaris M220 ultrasonicator and bar-coded using Ovation Ultralow Library Systems (Nugen). Samples were sequenced using an Illumina HiSeq 4000 by running a single-read 50-base-pair protocol (Cancer Research UK, Cambridge Institute). Sequence data were trimmed to remove adaptors and sequences with a quality score below 30 using Trim Galore (v.0.50, Babraham Bioinformatics) and then aligned to the mouse genome (GRCm38) using STAR (v.2.6.0a); differential expression was calculated using DESeq2 (v.1.18.1).

### Bioinformatic identification of candidate ligands and receptor pairs

Gene lists for cytokines and cytokine receptors were obtained by downloading Gene Ontology gene lists for ‘Cytokine Activity’ and ‘Cytokine Receptor Activity’ from the Mouse Genome Informatics website. The curated mouse CellTalkDB database of ligand–receptor pairs was used to identify interacting gene pairs between these gene lists^61^. Using R programming language, the dplyr package was utilized to filter the CellTalkDB database by the cytokine and cytokine receptor gene lists to remove non-cytokine-related ligand–receptor pairs. This filtered list of ligand–receptor pairs was then used to interrogate bulk RNA-seq data of pDCs and ILC2s isolated from mouse lung. Expression of cytokine ligand–receptor pairs in which expression of the receptor by pDCs was greater than 10 RPMK and expression of the ligand by ILC2s was greater than 10 RPKM was then extracted. Ligand–receptor pairs involving *Cd44* or *Cd74* were excluded from the analysis due to the high expression levels of these transcripts.

### Histology

Tissue was fixed in 10% formalin overnight and paraffin embedded; sections were stained with haematoxylin and eosin. Lung histology sections were assessed by a researcher blinded to groupings and given a score between 0 and 5 based on the presence or absence of large cellular aggregates.

### Microscopy

Mice were euthanized and received intracardiac perfusion with PBS, followed by 4% PFA (Invitrogen). Lung and LN were collected and fixed with 4% PFA overnight. Fixed tissues were washed with PBS and placed in 30% sucrose for 24 h. Subsequently these were embedded in Optimum Cutting Temperature compound (VWR, catalogue no. 25608-930), frozen in Isopentane and sectioned on a Leica CM1860 cryostat. Sections were incubated in a blocking solution (2% goat serum and 0.5% Triton X-100 in PBS) for 1 h at room temperature. Tissue sections were then incubated overnight at 4 °C with primary antibodies against CD3e, B220 and VEGFR3, then with secondary antibodies for 1 h at room temperature. Images were acquired with an Olympus VS200 slide scanner and processed and analysed using ImageJ2 v.2.14.0/1.5 f.

Lung histology sections were assessed for TLS by a researcher blinded to groupings and given a score between 0 and 5 based on the pathology.

### Data and statistical analyses

Statistical analysis was performed using GraphPad Prism v.10.0b software.

Bulk RNA-seq data generated in this study have been deposited at the Gene Expression Omnibus (GEO) under accession number GSE243691.

### Reporting summary

Further information on research design is available in the Nature Portfolio Reporting Summary linked to this article.

## Online content

Any methods, additional references, Nature Portfolio reporting summaries, source data, extended data, supplementary information, acknowledgements, peer review information; details of author contributions and competing interests; and statements of data and code availability are available at 10.1038/s41586-024-07746-w.

### Supplementary information

Reporting Summary

### Source data

Source Data Figs. 1–5 and Extended Data Figs. 1–9

## Figures and Tables

**Extended Data Table 1:**
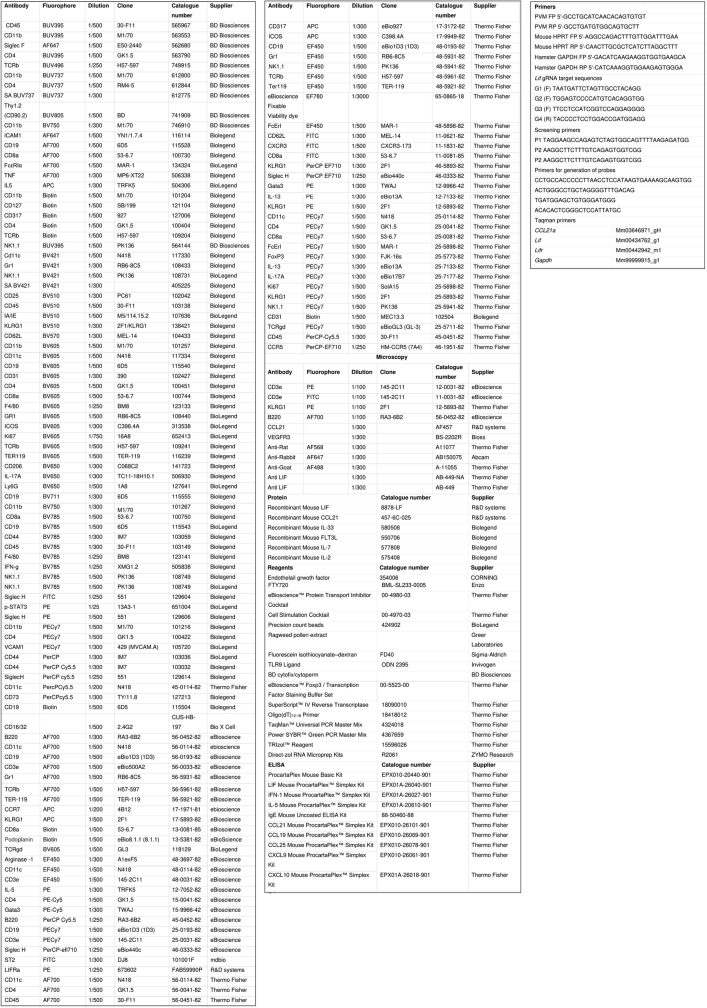
Reagent list

## Data Availability

All high-throughput data in this study were deposited at Gene Expression Omnibus under accession no. GSE243691 Source data are provided with this paper.
