# Supplementary material for: ILC2-derived LIF licences progress from tissue to systemic immunity
Source: Nature. 2024 Aug 7;632(8026):885–92. doi: 10.1038/s41586-024-07746-w (PMC11338826; doi:10.1038/s41586-024-07746-w)
Supplement: Supplementary file 1 — Reporting Summary [file 41586_2024_7746_MOESM1_ESM.pdf]

nature portfolio

Corresponding author(s): Andrew McKenzie
Mayuri Gogoi

Last updated by author(s): May 21, 2024

Reporting Summary

Nature Portfolio wishes to improve the reproducibility of the work that we publish. This form provides structure for consistency and transparency in reporting. For further information on Nature Portfolio policies, see our Editorial Policies and the Editorial Policy Checklist.

Statistics

For all statistical analyses, confirm that the following items are present in the figure legend, table legend, main text, or Methods section.

n/a	Confirmed	
☐	☒	The exact sample size (*n*) for each experimental group/condition, given as a discrete number and unit of measurement
☐	☒	A statement on whether measurements were taken from distinct samples or whether the same sample was measured repeatedly
☐	☒	The statistical test(s) used AND whether they are one- or two-sided<br>*Only common tests should be described solely by name; describe more complex techniques in the Methods section.*
☒	☐	A description of all covariates tested
☒	☐	A description of any assumptions or corrections, such as tests of normality and adjustment for multiple comparisons
☐	☒	A full description of the statistical parameters including central tendency (e.g. means) or other basic estimates (e.g. regression coefficient) AND variation (e.g. standard deviation) or associated estimates of uncertainty (e.g. confidence intervals)
☐	☒	For null hypothesis testing, the test statistic (e.g. *F*, *t*, *r*) with confidence intervals, effect sizes, degrees of freedom and *P* value noted<br>*Give P values as exact values whenever suitable.*
☒	☐	For Bayesian analysis, information on the choice of priors and Markov chain Monte Carlo settings
☒	☐	For hierarchical and complex designs, identification of the appropriate level for tests and full reporting of outcomes
☒	☐	Estimates of effect sizes (e.g. Cohen's *d*, Pearson's *r*), indicating how they were calculated

Our web collection on statistics for biologists contains articles on many of the points above.

Software and code

Policy information about availability of computer code

Data collection	BD LSRFortessa Special Order (5 laser), BD<br>BD FACSDiva software V6.2<br>ID7000 Spectral Cell Analyser (Sony)<br>iCyt Synergy, Sony Biotechnology SY3200<br>Illumina Hiseq4000<br>Olympus VS200 slide scanner<br>Luminex MAGPIX
Data analysis	RNA-seq: Sequence data were trimmed to remove adaptors and sequences with a quality score below 30 using Trim Galore (version 0.50, Babraham Bioinformatics) and then aligned to the mouse genome (GRCm38) using STAR (version 2.6.0a), and differential expression was calculated using DESeq2 (version 1.18.1).<br><br>Prism 9, GraphPad Prism<br>FlowJo. FlowJo, LLC, v10, RRID: SCR_008520<br>ImageJ2. Verson:2.14.0/1.5f

For manuscripts utilizing custom algorithms or software that are central to the research but not yet described in published literature, software must be made available to editors and reviewers. We strongly encourage code deposition in a community repository (e.g. GitHub). See the Nature Portfolio guidelines for submitting code & software for further information.

Data

Policy information about availability of data

All manuscripts must include a data availability statement. This statement should provide the following information, where applicable:

- Accession codes, unique identifiers, or web links for publicly available datasets
- A description of any restrictions on data availability
- For clinical datasets or third party data, please ensure that the statement adheres to our policy

All high-throughput data in this study were deposited at the Gene Expression Omnibus (GEO) under accession number GSE243691

Research involving human participants, their data, or biological material

Policy information about studies with human participants or human data. See also policy information about sex, gender (identity/presentation), and sexual orientation and race, ethnicity and racism.

Reporting on sex and gender	N/A
Reporting on race, ethnicity, or other socially relevant groupings	N/A
Population characteristics	N/A
Recruitment	N/A
Ethics oversight	N/A

Note that full information on the approval of the study protocol must also be provided in the manuscript.

Field-specific reporting

Please select the one below that is the best fit for your research. If you are not sure, read the appropriate sections before making your selection.

☒ Life sciences ☐ Behavioural & social sciences ☐ Ecological, evolutionary & environmental sciences

For a reference copy of the document with all sections, see nature.com/documents/nr-reporting-summary-flat.pdf

Life sciences study design

All studies must disclose on these points even when the disclosure is negative.

Sample size	Information on sample size is provided within each figure legend.<br>No statistical methods were used to predetermine sample size. Sample size were determined by prior experience as mentioned in the following articles<br>1 Kerscher, B. et al. BET Bromodomain Inhibitor iBET151 Impedes Human ILC2 Activation and Prevents Experimental Allergic Lung Inflammation. Front Immunol 10, 678 (2019). https://doi.org:10.3389/fimmu.2019.00678<br>2 Szeto, A. C. H. et al. An alphavbeta3 integrin checkpoint is critical for efficient T(H)2 cell cytokine polarization and potentiation of antigen-specific immunity. Nat Immunol 24, 123-135 (2023). https://doi.org:10.1038/s41590-022-01378-w<br>3 Panova, V. et al. Group-2 innate lymphoid cell-dependent regulation of tissue neutrophil migration by alternatively activated macrophage-secreted Ear11. Mucosal Immunol 14, 26-37 (2021). https://doi.org:10.1038/s41385-020-0298-2
Data exclusions	No data were excluded from analysis of in vitro experiments.<br><br>For in vivo experiments, outliers may result from variability in the technical treatment and subsequent suboptimal induction of inflammation. The control groups (PBS/Naive) provide a baseline comparison and are included in our experiments for this purpose. Outliers were identified using the ROUT method in PRISM. Once identified, all parameters from the outlier samples were excluded from analysis.
Replication	All experiments were replicated in at least 2 independent experiments using biologically independent samples (individual mice) within each experiment. All attempts at replication were successful.
Randomization	Sex- and aged- matched control and experimental mice were used in in vivo experiments according to obtained genotypes. Mice were randomly allocated in groups according to their genotypes for both in vivo and in vitro experiments .
Blinding	Investigators were blinded to group allocation during data collection and analysis.

Reporting for specific materials, systems and methods

We require information from authors about some types of materials, experimental systems and methods used in many studies. Here, indicate whether each material, system or method listed is relevant to your study. If you are not sure if a list item applies to your research, read the appropriate section before selecting a response.

Materials & experimental systems

n/a	Involved in the study
☐	☒ Antibodies
☐	☒ Eukaryotic cell lines
☒	☐ Palaeontology and archaeology
☐	☒ Animals and other organisms
☒	☐ Clinical data
☒	☐ Dual use research of concern
☒	☐ Plants

Methods

n/a	Involved in the study
☒	☐ ChIP-seq
☐	☒ Flow cytometry
☒	☐ MRI-based neuroimaging

Antibodies

Antibodies used

Flowcytometry:
Antibody, Fluorophore, Dilution, Clone, Catalogue number, Supplier
CD16/32 1/500 2.4G2 CUS-HB-197 Bio X Cell
CD45 BUV395 1/500 30-F11 565967 BD Biosciences
CD11b BUV395 1/500 M1/70 563553 BD Biosciences
Siglec F AF647 1/500 E50-2440 562680 BD Biosciences
CD4 BUV395 1/500 GK1.5 563790 BD Biosciences
TCRb BUV496 1/250 H57-597 749915 BD Biosciences
CD11b BUV737 1/500 M1/70 612800 BD Biosciences
CD4 BUV737 1/500 RM4-5 612844 BD Biosciences
SA BUV737 BUV737 1/300 612775 BD Biosciences
Thy1.2 (CD90.2) BUV805 1/500 BD 741909 BD Biosciences
CD11b BV750 1/300 M1/70 746910 BD Biosciences
ICAM1 AF647 1/500 YN1/1.7.4 116114 Biolegend
CD19 AF700 1/500 6D5 115528 Biolegend
CD8a AF700 1/500 53-6.7 100730 Biolegend
FcεRIα AF700 1/500 MAR-1 134324 BioLegend
TNF AF700 1/300 MP6-XT22 506338 Biolegend
CD45 APC 0.3 ug in 200 ul 30-F11 17-0451-82 Thermo Fisher
IL5 APC 1/300 TRFK5 504306 BioLegend
CD11b Biotin 1/500 M1/70 101204 Biolegend
CD127 Biotin 1/500 SB/199 121104 Biolegend
CD317 Biotin 1/500 927 127006 Biolegend
CD4 Biotin 1/500 GK1.5 100404 Biolegend
TCRb Biotin 1/500 H57-597 109204 Biolegend
NK1.1 BUV395 1/500 PK136 564144 BD Biosciences
Cd11c BV421 1/500 N418 117330 Biolegend
Gr1 BV421 1/300 RB6-8C5 108433 Biolegend
NK1.1 BV421 1/500 PK136 108731 BioLegend
SA BV421 BV421 1/300 405225 Biolegend
CD25 BV510 1/300 PC61 102042 Biolegend
CD45 BV510 1/500 30-F11 103138 Biolegend
IA/IE BV510 1/500 M5/114.15.2 107636 BioLegend
KLRG1 BV510 1/300 2F1/KLRG1 138421 Biolegend
CD62L BV570 1/300 MEL-14 104433 Biolegend
CD11b BV605 1/500 M1/70 101257 Biolegend
CD11c BV605 1/500 N418 117334 Biolegend
CD19 BV605 1/500 6D5 115540 Biolegend
CD31 BV605 1/300 390 102427 Biolegend
CD4 BV605 1/500 GK1.5 100451 Biolegend
CD8a BV605 1/500 53-6.7 100744 Biolegend
F4/80 BV605 1/250 BM8 123133 Biolegend
GR1 BV605 1/500 RB6-8C5 108440 BioLegend
ICOS BV605 1/300 C398.4A 313538 BioLegend
Ki67 BV605 1/750 16A8 652413 BioLegend
TCRb BV605 1/500 H57-597 109241 Biolegend
TER119 BV605 1/500 TER-119 116239 Biolegend
CD206 BV650 1/300 C068C2 141723 Biolegend
IL-17A BV650 1/300 TC11-18H10.1 506930 Biolegend
Ly6G BV650 1/500 1A8 127641 BioLegend

CD19 BV711 1/300 6D5 115555 Biolegend
CD11b BV750 1/300 M1/70 101267 Biolegend
CD8a BV785 1/500 53-6.7 100750 Biolegend
CD19 BV785 1/500 6D5 115543 BioLegend
CD44 BV785 1/300 IM7 103059 Biolegend
CD45 BV785 1/300 30-F11 103149 Biolegend
F4/80 BV785 1/250 BM8 123141 Biolegend
IFN-g BV785 1/250 XMG1.2 505838 Biolegend
NK1.1 BV785 1/500 PK136 108749 Biolegend
NK1.1 BV785 1/500 PK136 108749 BioLegend
Siglec H FITC 1/250 551 129604 Biolegend
p-STAT3 PE 1/25 13A3-1 651004 BioLegend
Siglec H PE 1/500 551 129606 Biolegend
CD11b PECy7 1/500 M1/70 101216 Biolegend
CD4 PECy7 1/500 GK1.5 100422 Biolegend
VCAM1 PECy7 1/300 429 (MVCAM.A) 105720 BioLegend
CD44 PerCP 1/300 IM7 103036 BioLegend
CD44 PerCP Cy5.5 1/300 IM7 103032 Biolegend
SiglecH PerCP cy5.5 1/250 551 129614 Biolegend
CD11c PercPCy5.5 1/200 N418 45-0114-82 Thermo Fisher
CD73 PerCPcy5.5 1/300 TY/11.8 127213 Biolegend
CD19 Biotin 1/500 6D5 115504 Biolegend
B220 AF700 1/300 RA3-6B2 56-0452-82 eBioscience
CD11c AF700 1/500 N418 56-0114-82 ebioscience
CD19 AF700 1/500 eBio1D3 (1D3) 56-0193-82 eBioscience
CD3e AF700 1/500 eBio500A2 56-0033-82 eBioscience
Gr1 AF700 1/500 RB6-8C5 56-5931-82 eBioscience
TCRb AF700 1/500 H57-597 56-5961-82 eBioscience
TER-119 AF700 1/500 TER-119 56-5921-82 eBioscience
CCR7 APC 1/200 4B12 17-1971-81 ebioscience
KLRG1 APC 1/500 2F1 17-5893-82 eBioscience
CD8a Biotin 1/500 53-6.7 13-0081-85 eBioscience
Podoplanin Biotin 1/500 eBio8.1.1 (8.1.1) 13-5381-82 eBioScience
TCRgd BV605 1/500 GL3 118129 BioLegend
Arginase -1 EF450 1/300 A1exF5 48-3697-82 eBioscience
CD11c EF450 1/500 N418 48-0114-82 eBioscience
CD3e EF450 1/500 145-2C11 48-0031-82 eBioscience
IL-5 PE 1/300 TRFK5 12-7052-82 eBioscience
CD4 PE-Cy5 1/500 GK1.5 15-0041-82 eBioscience
Gata3 PE-Cy5 1/300 TWAJ 15-9966-42 eBioscience
B220 PerCP Cy5.5 1/250 RA3-6B2 45-0452-82 eBioscience
CD19 PECy7 1/500 eBio1D3 (1D3) 25-0193-82 eBioscience
CD3e PECy7 1/500 145-2C11 25-0031-82 eBioscience
Siglec H   PerCP-efl710 1/250 eBio440c 46-0333-82 eBioscience
ST2 FITC 1/300 DJ8 101001F mdbio
LIFRa PE 1/250 673602 FAB59990P R&D systems
CD11c AF700 1/500 N418 56-0114-82 Thermo Fisher
CD4 AF700 1/500 GK1.5 56-0041-82 Thermo Fisher
CD45 AF700 1/500 30-F11 56-0451-82 Thermo Fisher
CD317 APC 1/300 eBio927 17-3172-82 Thermo Fisher
ICOS APC 1/300 C398.4A 17-9949-82 Thermo Fisher
CD19 EF450 1/500 eBio1D3 (1D3) 48-0193-82 Thermo Fisher
Gr1 EF450 1/500 RB6-8C5 48-5931-82 Thermo Fisher
NK1.1 EF450 1/500 PK136 48-5941-82 Thermo Fisher
TCRb EF450 1/500 H57-597 48-5961-82 Thermo Fisher
Ter119 EF450 1/500 TER-119 48-5921-82 Thermo Fisher
eBioscience Fixable Viability dye EF780 1/3000   65-0865-18 Thermo Fisher
FcErI EF450 1/500 MAR-1 48-5898-82 Thermo Fisher
CD62L FITC 1/300 MEL-14 11-0621-82 Thermo Fisher
CXCR3 FITC 1/500 CXCR3-173 11-1831-82 Thermo Fisher
CD8a FITC 1/300 53-6.7 11-0081-85 Thermo Fisher
KLRG1 PerCP EF710 1/300 2F1 46-5893-82 Thermo Fisher
Siglec H PerCP EF710 1/250 eBio440c 46-0333-82 Thermo Fisher
Gata3 PE 1/300 TWAJ 12-9966-42 Thermo Fisher
IL-13 PE 1/300 eBio13A 12-7133-82 Thermo Fisher
KLRG1 PE 1/500 2F1 12-5893-82 Thermo Fisher
CD11c PECy7 1/500 N418 25-0114-82 Thermo Fisher
CD4 PECy7 1/500 GK1.5 25-0041-82 Thermo Fisher
CD8a PECy7 1/500 53-6.7 25-0081-82 Thermo Fisher
FcErI PECy7 1/500 MAR-1 25-5898-82 Thermo Fisher
FoxP3 PECy7 1/300 FJK-16s 25-5773-82 Thermo Fisher
IL-13 PECy7 1/300 eBio13A 25-7133-82 Thermo Fisher
IL-17A PECy7 1/300 eBio17B7 25-7177-82 Thermo Fisher
Ki67 PECy7 1/500 SolA15 25-5698-82 Thermo Fisher
KLRG1 PECy7 1/500 2F1 25-5893-82 Thermo Fisher
NK1.1 PECy7 1/500 PK136 25-5941-82 Thermo Fisher

CD31 Biotin 1/500 MEC13.3 102504 Biolegend
TCRgd PECy7 1/500 eBioGL3 (GL-3) 25-5711-82 Thermo Fisher
CD45 PerCP-Cy5.5 1/300 30-F11 45-0451-82 Thermo Fisher
CCR5 PerCP-EF710 1/250 HM-CCR5 (7A4) 46-1951-82 Thermo Fisher

Microscopy
Antibody Fluorophore Dilution Clone Catalogue number Supplier
CD3e PE 1/100 145-2C11 12-0031-82 eBioscience
CD3e FITC 1/100 145-2C11 11-0031-82 eBioscience
KLRG1 PE 1/100 2F1 12-5893-82 Thermo Fisher
B220 AF700 1/100 RA3-6B2 56-0452-82 eBioscience
VEGFR3   1/300   BS-2202R Bioss
Anti-Rat AF568 1/300   A11077 Thermo Fisher
Anti-Rabbit AF647 1/300   AB150075 Abcam
Anti-Goat AF488 1/300   A-11055 Thermo Fisher

Validation

All used ELISA kits and antibodies are commercially available and have been validated by the manufacturer. Validations and detail product information are available on thewebsites:
Flowcytometry:
https://bioxcell.com/recombimab-anti-mouse-cd16-cd32-cp025
https://www.bdbiosciences.com/en-ca/products/reagents/flow-cytometry-reagents/research-reagents/single-color-antibodies-ruo/buv395-rat-anti-mouse-cd45.565967
https://www.bdbiosciences.com/en-ca/products/reagents/flow-cytometry-reagents/research-reagents/single-color-antibodies-ruo/buv395-rat-anti-cd11b.565976
https://www.bdbiosciences.com/en-ca/products/reagents/flow-cytometry-reagents/research-reagents/single-color-antibodies-ruo/alexa-fluor-647-rat-anti-mouse-siglec-f.562680
https://www.bdbiosciences.com/en-ca/products/reagents/flow-cytometry-reagents/research-reagents/single-color-antibodies-ruo/buv395-rat-anti-mouse-cd4.563790
https://www.bdbiosciences.com/en-ca/products/reagents/flow-cytometry-reagents/research-reagents/single-color-antibodies-ruo/buv496-hamster-anti-mouse-tcr-chain.749915
https://www.bdbiosciences.com/en-ca/products/reagents/flow-cytometry-reagents/research-reagents/single-color-antibodies-ruo/buv737-rat-anti-cd11b.741722
https://www.bdbiosciences.com/en-ca/products/reagents/flow-cytometry-reagents/research-reagents/single-color-antibodies-ruo/buv737-rat-anti-mouse-cd4.612844
https://www.bdbiosciences.com/en-ca/products/reagents/flow-cytometry-reagents/research-reagents/single-color-antibodies-ruo/buv737-streptavidin.612775
https://www.bdbiosciences.com/en-ca/products/reagents/flow-cytometry-reagents/research-reagents/single-color-antibodies-ruo/buv805-rat-anti-mouse-cd90-2.741909
https://www.bdbiosciences.com/en-ca/products/reagents/flow-cytometry-reagents/research-reagents/single-color-antibodies-ruo/bv750-rat-anti-cd11b.746910
https://www.biolegend.com/en-gb/products/alexa-fluor-647-anti-mouse-cd54-antibody-3110
https://www.biolegend.com/en-gb/products/alexa-fluor-700-anti-mouse-cd19-antibody-3391
https://www.biolegend.com/en-gb/products/alexa-fluor-700-anti-mouse-cd8a-antibody-3387
https://www.biolegend.com/en-gb/products/alexa-fluor-700-anti-mouse-fcepsilonrialpha-antibody-12817
https://www.biolegend.com/en-gb/products/alexa-fluor-700-anti-mouse-tnf-alpha-antibody-9146
https://www.thermofisher.com/antibody/product/CD45-Antibody-clone-30-F11-Monoclonal/17-0451-82
https://www.biolegend.com/en-gb/products/apc-anti-mouse-human-il-5-antibody-989
https://www.biolegend.com/en-gb/products/biotin-anti-mouse-human-cd11b-antibody-346
https://www.biolegend.com/en-gb/products/biotin-anti-mouse-cd127-il-7ralpha-antibody-3048

https://www.biolegend.com/en-gb/products/biotin-anti-mouse-cd317-bst2-pdca-1-antibody-6348
https://www.biolegend.com/en-gb/products/biotin-anti-mouse-cd4-antibody-247
https://www.biolegend.com/en-gb/products/biotin-anti-mouse-tcr-beta-chain-antibody-269
https://www.bdbiosciences.com/en-ca/products/reagents/flow-cytometry-reagents/research-reagents/single-color-antibodies-ruo/buv395-mouse-anti-mouse-nk-1-1.564144
https://www.biolegend.com/en-gb/products/brilliant-violet-421-anti-mouse-cd11c-antibody-7149
https://www.biolegend.com/en-gb/products/brilliant-violet-421-anti-mouse-ly-6g-ly-6c-gr-1-antibody-7201
https://www.biolegend.com/en-gb/products/brilliant-violet-421-anti-mouse-nk-1-1-antibody-7150
https://www.biolegend.com/en-gb/products/brilliant-violet-421-streptavidin-7297
https://www.biolegend.com/en-gb/products/brilliant-violet-510-anti-mouse-cd25-antibody-8663
https://www.biolegend.com/en-gb/products/brilliant-violet-510-anti-mouse-cd45-antibody-7995
https://www.biolegend.com/en-gb/products/brilliant-violet-510-anti-mouse-i-a-i-e-antibody-7997
https://www.biolegend.com/en-gb/products/brilliant-violet-510-anti-mouse-human-klrg1-mafa-antibody-9943
https://www.biolegend.com/en-gb/products/brilliant-violet-570-anti-mouse-cd62l-antibody-7369
https://www.biolegend.com/en-gb/products/brilliant-violet-605-anti-mouse-human-cd11b-antibody-7637
https://www.biolegend.com/en-gb/products/brilliant-violet-605-anti-mouse-cd11c-antibody-7865
https://www.biolegend.com/en-gb/products/brilliant-violet-605-anti-mouse-cd19-antibody-7645
https://www.biolegend.com/en-gb/products/brilliant-violet-605-anti-mouse-cd31-antibody-9963
https://www.biolegend.com/en-gb/products/brilliant-violet-605-anti-mouse-cd4-antibody-10708
https://www.biolegend.com/en-gb/products/brilliant-violet-605-anti-mouse-cd8a-antibody-7636
https://www.biolegend.com/en-gb/products/brilliant-violet-605-anti-mouse-f4-80-antibody-8702
https://www.biolegend.com/en-gb/products/brilliant-violet-605-anti-mouse-ly-6g-ly-6c-gr-1-antibody-8724
https://www.biolegend.com/en-gb/products/brilliant-violet-605-anti-human-mouse-rat-cd278-icos-antibody-14371
https://www.biolegend.com/en-us/products/brilliant-violet-605-anti-mouse-tcr-beta-chain-antibody-13533

https://www.biolegend.com/en-us/products/brilliant-violet-605-anti-mouse-ter-119-erythroid-cells-antibody-8839
https://www.biolegend.com/en-us/products/brilliant-violet-650-anti-mouse-cd206-mmr-antibody-8842
https://www.biolegend.com/en-us/products/brilliant-violet-650-anti-mouse-il-17a-antibody-7684
https://www.biolegend.com/en-us/products/brilliant-violet-650-anti-mouse-ly-6g-antibody-11981
https://www.biolegend.com/en-us/products/brilliant-violet-711-anti-mouse-cd19-antibody-12075
https://www.biolegend.com/en-us/products/brilliant-violet-750-anti-mousehuman-cd11b-antibody-17501
https://www.biolegend.com/en-us/products/brilliant-violet-785-anti-mouse-cd8a-antibody-7957
https://www.biolegend.com/en-us/products/brilliant-violet-785-anti-mouse-cd19-antibody-7962
https://www.biolegend.com/en-us/products/brilliant-violet-785-anti-mouse-cd19-antibody-7962
https://www.biolegend.com/en-us/products/brilliant-violet-785-anti-mouse-human-cd44-antibody-7959
https://www.biolegend.com/en-us/products/brilliant-violet-785-anti-mouse-cd45-antibody-10636
https://www.biolegend.com/en-us/products/brilliant-violet-785-anti-mouse-f4-80-antibody-9919
https://www.biolegend.com/en-us/products/brilliant-violet-785-anti-mouse-ifn-gamma-antibody-7987
https://www.biolegend.com/en-us/products/brilliant-violet-785-anti-mouse-nk-1-1-antibody-10367
https://www.biolegend.com/en-us/products/brilliant-violet-785-anti-mouse-nk-1-1-antibody-10367
https://www.biolegend.com/en-us/products/fitc-anti-mouse-siglec-h-antibody-5177
https://www.biolegend.com/en-us/products/pe-anti-stat3-phospho-tyr705-antibody-12914
https://www.biolegend.com/en-us/products/pe-anti-mouse-siglec-h-antibody-5178
https://www.biolegend.com/en-us/products/pe-cyanine7-anti-mouse-human-cd11b-antibody-1921
https://www.biolegend.com/en-us/products/pe-cyanine7-anti-mouse-cd4-antibody-1919
https://www.biolegend.com/en-us/products/pe-cyanine7-anti-mouse-cd106-antibody-6135
https://www.biolegend.com/en-us/products/percp-anti-mouse-human-cd44-antibody-6895
https://www.biolegend.com/en-us/products/percp-cyanine5-5-anti-mouse-human-cd44-antibody-5605
https://www.biolegend.com/en-us/products/percp-cyanine5-5-anti-mouse-siglec-h-antibody-6927
https://www.thermofisher.com/antibody/product/CD11c-Antibody-clone-N418-Monoclonal/45-0114-82
https://www.biolegend.com/en-us/products/percp-cyanine5-5-anti-mouse-cd73-antibody-7895
https://www.biolegend.com/en-us/products/biotin-anti-mouse-cd19-antibody-1527
https://www.thermofisher.com/antibody/product/CD45R-B220-Antibody-clone-RA3-6B2-Monoclonal/56-0452-82
https://www.thermofisher.com/antibody/product/CD11c-Antibody-clone-118-A5-Monoclonal/14-9761-82
https://www.thermofisher.com/antibody/product/CD11c-Antibody-clone-N418-Monoclonal/56-0114-82
https://www.thermofisher.com/antibody/product/CD19-Antibody-clone-eBio1D3-1D3-Monoclonal/56-0193-82
https://www.thermofisher.com/antibody/product/Ly-6G-Ly-6C-Antibody-clone-RB6-8C5-Monoclonal/56-5931-82
https://www.thermofisher.com/antibody/product/TCR-beta-Antibody-clone-H57-597-Monoclonal/56-5961-82
https://www.thermofisher.com/antibody/product/TER-119-Antibody-clone-TER-119-Monoclonal/56-5921-82
https://www.thermofisher.com/antibody/product/CD197-CCR7-Antibody-clone-4B12-Monoclonal/17-1971-82
https://www.thermofisher.com/antibody/product/KLRG1-Antibody-clone-2F1-Monoclonal/17-5893-82
https://www.thermofisher.com/antibody/product/CD8a-Antibody-clone-53-6-7-Monoclonal/13-0081-82
https://www.thermofisher.com/antibody/product/Podoplanin-Antibody-clone-eBio8-1-1-8-1-1-Monoclonal/13-5381-82
https://www.biolegend.com/en-gb/products/brilliant-violet-605-anti-mouse-tcr-gamma-delta-antibody-9655?GroupID=BLG3687
https://www.thermofisher.com/antibody/product/Arginase-1-Antibody-clone-A1exF5-Monoclonal/48-3697-82
https://www.thermofisher.com/antibody/product/CD11c-Antibody-clone-N418-Monoclonal/48-0114-82
https://www.thermofisher.com/antibody/product/CD3e-Antibody-clone-145-2C11-Monoclonal/48-0031-82
https://www.thermofisher.com/antibody/product/IL-5-Antibody-clone-TRFK5-Monoclonal/12-7052-82
https://www.thermofisher.com/antibody/product/CD4-Antibody-clone-GK1-5-Monoclonal/15-0041-82
https://www.thermofisher.com/antibody/product/Gata-3-Antibody-clone-TWAJ-Monoclonal/15-9966-42
https://www.thermofisher.com/antibody/product/CD45R-B220-Antibody-clone-RA3-6B2-Monoclonal/14-0452-82

https://www.thermofisher.com/antibody/product/CD19-Antibody-clone-eBio1D3-1D3-Monoclonal/25-0193-82
https://rnaidesigner.thermofisher.com/antibody/product/CD3e-Antibody-clone-145-2C11-Monoclonal/25-0031-82
https://rnaidesigner.thermofisher.com/antibody/product/SIGLEC-H-Antibody-clone-eBio440c-Monoclonal/46-0333-82
https://www.mdbioproducts.com/products/t1-st2-il-33-r-mouse-monoclonal-antibody?variant=39848199422141
https://www.rndsystems.com/products/mouse-lifralpha-pe-conjugated-antibody-673602_fab5990p
https://www.thermofisher.com/antibody/product/CD11c-Antibody-clone-N418-Monoclonal/56-0114-82
https://www.thermofisher.com/antibody/product/CD4-Antibody-clone-GK1-5-Monoclonal/56-0041-82
https://www.thermofisher.com/antibody/product/CD45-Antibody-clone-30-F11-Monoclonal/56-0451-82
https://tfcom-global-nginx.commerceprod.thermofisher.com/antibody/product/CD317-BST2-PDCA-1-Antibody-clone-eBio927-Monoclonal/17-3172-82
https://www.thermofisher.com/antibody/product/CD278-ICOS-Antibody-clone-C398-4A-Monoclonal/17-9949-82
https://www.thermofisher.com/antibody/product/CD19-Antibody-clone-eBio1D3-1D3-Monoclonal/48-0193-82
https://www.thermofisher.com/antibody/product/Ly-6G-Ly-6C-Antibody-clone-RB6-8C5-Monoclonal/48-5931-82
https://www.thermofisher.com/antibody/product/NK1-1-Antibody-clone-PK136-Monoclonal/48-5941-82
https://www.thermofisher.com/antibody/product/TCR-beta-Antibody-clone-H57-597-Monoclonal/48-5961-82
https://www.thermofisher.com/antibody/product/TER-119-Antibody-clone-TER-119-Monoclonal/48-5921-82
https://www.thermofisher.com/order/catalog/product/65-0865-18
https://www.thermofisher.com/antibody/product/FceR1-alpha-Antibody-clone-MAR-1-Monoclonal/48-5898-82
https://www.thermofisher.com/antibody/product/CD62L-L-Selectin-Antibody-clone-MEL-14-Monoclonal/11-0621-82
https://www.thermofisher.com/antibody/product/CD183-CXCR3-Antibody-clone-CXCR3-173-Monoclonal/11-1831-82
https://www.thermofisher.com/antibody/product/CD8a-Antibody-clone-53-6-7-Monoclonal/11-0081-82
https://www.thermofisher.com/antibody/product/KLRG1-Antibody-clone-2F1-Monoclonal/46-5893-82
https://rnaidesigner.thermofisher.com/antibody/product/SIGLEC-H-Antibody-clone-eBio440c-Monoclonal/46-0333-82
https://www.thermofisher.com/antibody/product/Gata-3-Antibody-clone-TWAJ-Monoclonal/12-9966-42
https://www.thermofisher.com/antibody/product/IL-13-Antibody-clone-eBio13A-Monoclonal/12-7133-82
https://rnaidesigner.thermofisher.com/antibody/product/KLRG1-Antibody-clone-2F1-Monoclonal/12-5893-82

https://www.thermofisher.com/antibody/product/CD11c-Antibody-clone-N418-Monoclonal/25-0114-82
https://www.thermofisher.com/antibody/product/CD4-Antibody-clone-GK1-5-Monoclonal/25-0041-82
https://www.thermofisher.com/antibody/product/CD8a-Antibody-clone-53-6-7-Monoclonal/25-0081-82
https://www.thermofisher.com/antibody/product/FceR1-alpha-Antibody-clone-MAR-1-Monoclonal/25-5898-82
https://www.thermofisher.com/antibody/product/FOXP3-Antibody-clone-FJK-16s-Monoclonal/25-5773-82
https://www.thermofisher.com/antibody/product/IL-13-Antibody-clone-eBio13A-Monoclonal/25-7133-82
https://www.thermofisher.com/antibody/product/IL-17A-Antibody-clone-eBio17B7-Monoclonal/25-7177-82
https://www.thermofisher.com/antibody/product/Ki-67-Antibody-clone-SolA15-Monoclonal/25-5698-82
https://www.thermofisher.com/antibody/product/KLRG1-Antibody-clone-2F1-Monoclonal/25-5893-82
https://www.thermofisher.com/antibody/product/NK1-1-Antibody-clone-PK136-Monoclonal/25-5941-82
https://www.biolegend.com/en-gb/search-results/biotin-anti-mouse-cd31-antibody-376
https://www.thermofisher.com/antibody/product/TCR-gamma-delta-Antibody-clone-eBioGL3-GL-3-GL3-Monoclonal/25-5711-82
https://www.thermofisher.com/antibody/product/CD45-Antibody-clone-30-F11-Monoclonal/45-0451-82
https://rnaidesigner.thermofisher.com/antibody/product/CD195-CCR5-Antibody-clone-HM-CCR5-7A4-Monoclonal/46-1951-82

Microscopy
https://www.thermofisher.com/antibody/product/CD3e-Antibody-clone-145-2C11-Monoclonal/12-0031-82
https://www.thermofisher.com/antibody/product/CD3e-Antibody-clone-145-2C11-Monoclonal/11-0031-82
https://rnaidesigner.thermofisher.com/antibody/product/KLRG1-Antibody-clone-2F1-Monoclonal/12-5893-82
https://www.thermofisher.com/antibody/product/CD45R-B220-Antibody-clone-RA3-6B2-Monoclonal/56-0452-82
https://www.biossantibodies.com/datasheets/bs-2202R
https://www.thermofisher.com/antibody/product/Goat-anti-Rat-IgG-H-L-Cross-Adsorbed-Secondary-Antibody-Polyclonal/A-11077
https://www.abcam.com/products/secondary-antibodies/donkey-rabbit-igg-hl-alexa-fluor-647-ab150075.html
https://www.thermofisher.com/antibody/product/Donkey-anti-Goat-IgG-H-L-Cross-Adsorbed-Secondary-Antibody-Polyclonal/A-11055

Eukaryotic cell lines

Policy information about cell lines and Sex and Gender in Research

Cell line source(s)	BHK-21 clone 13 cell line (immortalized cell line derived from hamster kidney)<br>RRID:CVCL_1915
Authentication	Cell lines were not authenticated but were uitiized within 10 passages of a master stock
Mycoplasma contamination	Not tested
Commonly misidentified lines<br>(See ICLAC register)	This cell line isn't listed on the ICLAC database

Animals and other research organisms

Policy information about studies involving animals; ARRIVE guidelines recommended for reporting animal research, and Sex and Gender in Research

Laboratory animals	C57BL/6JOla controls were bred in MRC-LMB. All mice were either on the C57BL/6J Ola background or back-crossed for at least six generations.<br>Il7raCre (Schlenner, S. M. et al. Fate mapping reveals separate origins of T cells and myeloid lineages in the thymus. Immunity 32, 426-436 (2010))<br>Roraflox/flox (Oliphant, C. J. et al. MHCII-mediated dialog between group 2 innate lymphoid cells and CD4(+) T cells potentiates type 2 immunity and promotes parasitic helminth expulsion. Immunity 41, 283-295 (2014))<br>Il1rl1–/– (Townsend, M. J., Fallon, P. G., Matthews, D. J., Jolin, H. E. & McKenzie, A. N. T1/ST2-deficient mice demonstrate the importance of T1/ST2 in developing primary T helper cell type 2 responses. J Exp Med 191, 1069-1076 (2000))<br>Rag2–/–<br>Rag2–/–Il2rgc–/– (Rag2–/–gc–/–)<br>Lif flox/flox,MRC-LMB, accompanying manuscript<br>BIC mice (Il13Dre, Cd28Vika, IcosCre), (Szeto, Clarke et al., Science, in press)<br>ILC2LIFKO mice were BIC Lif flox/flox mice MRC-LMB, accompanying manuscript<br>SiglechCre, Puttur, F. et al. Absence of Siglec-H in MCMV infection elevates interferon alpha production but does not enhance viral clearance. PLoS Pathog 9, e1003648 (2013)<br>Lifr–/flox, MGI:4841519<br><br>All mice were maintained in the Medical Research Council ARES animal facility under specific pathogen-free conditions, at 19-23°C, 45-65% humidity, with a 12-h light-dark cycle. Mice used in the experiments were between 8-16 weeks old. In individual experiments, mice were matched for age, sex and background strain.
Wild animals	The study did not involve wild animals.
Reporting on sex	Mice were sex matched for in vivo experiments (within one experiment, n=3-10 per group) and both male and female cohorts (2-3

Reporting on sex	independent experiments) were used in this study , for each reported findings. For in vitro experiments male and female mice were used.
Field-collected samples	The study did not involve samples collected from the field.
Ethics oversight	All experiments undertaken in this study were done so with the approval of the LMB Animal Welfare and Ethical Review Body (AWERB) and of the UK Home Office.

Note that full information on the approval of the study protocol must also be provided in the manuscript.

Plants

Seed stocks	*Report on the source of all seed stocks or other plant material used. If applicable, state the seed stock centre and catalogue number. If plant specimens were collected from the field, describe the collection location, date and sampling procedures.*
Novel plant genotypes	*Describe the methods by which all novel plant genotypes were produced. This includes those generated by transgenic approaches, gene editing, chemical/radiation-based mutagenesis and hybridization. For transgenic lines, describe the transformation method, the number of independent lines analyzed and the generation upon which experiments were performed. For gene-edited lines, describe the editor used, the endogenous sequence targeted for editing, the targeting guide RNA sequence (if applicable) and how the editor was applied.*
Authentication	*Describe any authentication procedures for each seed stock used or novel genotype generated. Describe any experiments used to assess the effect of a mutation and, where applicable, how potential secondary effects (e.g. second site T-DNA insertions, mosiacism, off-target gene editing) were examined.*

Flow Cytometry

Plots

Confirm that:

☒ The axis labels state the marker and fluorochrome used (e.g. CD4-FITC).

☒ The axis scales are clearly visible. Include numbers along axes only for bottom left plot of group (a 'group' is an analysis of identical markers).

☒ All plots are contour plots with outliers or pseudocolor plots.

☒ A numerical value for number of cells or percentage (with statistics) is provided.

Methodology

Sample preparation	Tissue preparation. Lung tissue was predigested with 750 U ml-1 collagenase I (Gibco) and 0.3 mg ml-1 DNaseI (Sigma-Aldrich) before obtaining a single-cell suspension at 37 °C for 30 min; the tissue was passed through a 70 µm cell strainer. For lymphocyte enrichment, lung single-cell suspension was centrifuged through 30% Percoll (GE Healthcare) at 800 x g for 15 min. Spleen, thymus and mediastinal lymph node single cell suspensions were prepared by passing the tissue through a 70 µM cell strainer and lysing RBCs. Single bone marrow cell suspensions were prepared by flushing the femur and tibia with endotoxin-free PBS and lysing RBCs.
Instrument	ID7000 spectral cell analyser (Sony) LSRFortessa system (BD Biosciences) for analysis, iCyt Synergy system (70-um nozzle, Sony Biotechnology) for cell sorting.
Software	FACSDiva sofware (version 6.2, BD Biosciences)<br>FlowJo. FlowJo, LLC, v10, RRID: SCR_008520
Cell population abundance	Purity of sorted populations is typically >97%, as determined by analysis of sorted cells by flow cytometry.
Gating strategy	Cells are defined in FSC/SCC plot, followed by doublet exclusion in FCS-A/FCS-H plot. Positive populations are defined by comparison to unstained controls, isotype controls or fluorescence minus one controls.

☒ Tick this box to confirm that a figure exemplifying the gating strategy is provided in the Supplementary Information.
